# NMDA receptors in the lateral preoptic hypothalamus are essential for sustaining NREM and REM sleep

**DOI:** 10.1523/JNEUROSCI.0350-21.2022

**Published:** 2022-06-01

**Authors:** Giulia Miracca, Berta Anuncibay-Soto, Kyoko Tossell, Raquel Yustos, Alexei L. Vyssotski, Nicholas P. Franks, William Wisden

**Affiliations:** 1 Department of Life Sciences, Imperial College London, London SW7 2AZ, U.K; 2 UK Dementia Research Institute, Imperial College London, London SW7 2AZ, U.K; 3 Institute of Neuroinformatics, University of Zürich/ETH Zürich, Winterthurerstrasse 190, CH-8057, Zürich, Switzerland

## Abstract

The lateral preoptic (LPO) hypothalamus is a center for NREM and REM sleep induction and NREM sleep homeostasis. Although LPO is needed for NREM sleep, we found that calcium signals were, surprisingly, highest in REM sleep. Furthermore, and equally surprising, NMDA receptors in LPO were the main drivers of excitation. Deleting the NMDA receptor GluN1 subunit from LPO abolished calcium signals in all cells and produced insomnia. Mice of both sexes had highly fragmented NREM sleep-wake patterns and could not generate conventionally classified REM sleep. The sleep phenotype produced by deleting NMDA receptors depended on where in the hypothalamus the receptors were deleted. Deleting receptors from the anterior hypothalamic area did not influence sleep-wake states. The sleep fragmentation originated from NMDA receptors on GABA neurons in LPO. Sleep fragmentation could be transiently overcome with sleeping medication (zolpidem) or sedatives (dexmedetomidine). By contrast, fragmentation persisted under high sleep pressure produced by sleep deprivation - mice had a high propensity to sleep but woke up. By analyzing changes in delta power, sleep homeostasis (also referred to as “sleep drive”) remained intact after NMDA receptor ablation. We suggest NMDA glutamate receptor activation stabilizes firing of sleep-on neurons, and that mechanisms of sleep maintenance differ from that of the sleep drive itself.

## Introduction

Both NREM and REM sleep are partly controlled by the preoptic (PO) hypothalamus (Nauta, 1946; McGinty and Sterman, 1968; Sherin et al., 1996; John and Kumar, 1998; Lu et al., 2000; Lu et al., 2002; Szymusiak et al., 2007). In this region, GABA/peptidergic neurons, *e.g.* GABA/galanin neurons, contribute to NREM sleep induction and sleep homeostasis (Sherin et al., 1996; Zhang et al., 2015; Chung et al., 2017; Kroeger et al., 2018; Ma et al., 2019; Reichert et al., 2019). To stay asleep, it seems reasonable to assume that these sleep-promoting neurons would have to stay “on”. Indeed, lesioning of lateral PO (LPO) neurons in rats reduces the amounts of NREM or REM sleep, depending on the location of the lesion (Lu et al., 2000). But molecular factors that keep LPO sleep-promoting neurons firing and so govern the lengths of NREM and REM sleep episodes are not known.

One critical factor maintaining sleep could be NMDA-type glutamate receptors. These channels, especially when located extrasynaptically, can provide tonic excitation (Sah et al., 1989; Papouin et al., 2012; Neupane et al., 2021). Indeed, NMDA receptor activation promotes sleep. In the fruit fly *Drosophila,* genetic knockdown of NMDA receptors in brain reduces total sleep time (Tomita et al., 2015). In rodents, NMDA receptor antagonists reduce *and* agonists enhance NREM sleep (Tatsuki et al., 2016; Burgdorf et al., 2019). Furthermore, patients with autoimmunity to the essential GluN1 subunit of NMDA receptors often suffer insomnia (Dalmau et al., 2019; Arino et al., 2020). Because NMDA receptors are expressed throughout the brain (Moriyoshi et al., 1991; Laurie and Seeburg, 1994; Monyer et al., 1994), these effects on sleep could come from interference with many circuits.

Calcium entry through NMDA receptors has also been suggested to be part of the sleep homeostasis mechanism that tracks time spent awake (Liu et al., 2016). Even if sleep is poor, wakefulness still cannot be sustained beyond a certain limit. This limit is thought to be imposed by the process of sleep homeostasis, the increasing drive to enter NREM sleep as wakefulness continues (Borbely et al., 2016). Sleep homeostasis is operationally studied as an increase in NREM delta power after sleep deprivation (Hanlon et al., 2011; Franken, 2013; Greene et al., 2017; Deboer, 2018). Given that the PO hypothalamus is one of the key regions controlling sleep homeostasis (Zhang et al., 2015; Donlea et al., 2017; Ma et al., 2019; Reichert et al., 2019), we were keen to test how NMDA receptors in the LPO area influence this process.

We found that the whole LPO area has selectively raised calcium activity in REM sleep, and this calcium entry depends on NMDA receptors. In this study we deleted the GluN1 NMDA receptor subunit in the LPO hypothalamus and obtained a marked “insomnia” phenotype with high NREM sleep-wake fragmentation and greatly diminished REM sleep. Sleep homeostasis, however, was unaffected by removing NMDA receptors from LPO. The NREM sleep-wake fragmentation effect is selective for GluN1 expression in GABA neurons.

## Materials and Methods

### Mice

All experiments were performed in accordance with the United Kingdom Home Office Animal Procedures Act (1986) and were approved by the Imperial College Ethical Review Committee. Wild-type *C57BL/6J* mice were purchased from Charles River at 7/8 weeks of age. *Grin1^lox^* mice (Tsien et al., 1996) were purchased from The Jackson Laboratory (JAX stock number 005246) after kind donation by S. Tonegawa. *Vglut2-Cre* mice (*Vglut2-ires-Cre: Slc17a6^tm2(cre)Lowl^*/J) and *Vgat-Cre* mice (*Vgat-ires-Cre: Slc32a1^tm2(cre)Lowl^*/J) were kindly provided by B.B. Lowell and purchased from The Jackson Laboratory (JAX stock 016963 and 016962 respectively)(Vong et al., 2011). *Galanin-Cre* mice (*Tg(Gal-cre)KI87Gsat/Mmucd*) were generated by GENSAT and deposited at the Mutant Mouse Regional Resource Center, stock No. 031060-UCD, GENSAT- Project (NINDS Contracts N01NS02331 & HHSN271200723701C to The Rockefeller University, New York) (Schmidt et al., 2013). *Nos1-Cre* mice (*Nos1-ires-Cre^tm1(cre)Mgmj^*/J), were kindly provided by M. G. Myers, and purchased from The Jackson Laboratory (JAX stock 017526) (Leshan et al., 2012).

All mice were housed at a maximum of five mice per cage with food and water *ad libitum* and maintained under the same conditions (21±1 °C, reversed 12/12h dark/light cycle). Zeitgeber time (ZT) 0 is defined as the time when the light was switched on (16:00) and ZT12 is defined as the time of light off (4:00). For behavioral experiments, mice were singly housed, and experiments performed during dark phase (ZT12-24) unless otherwise specified, while photometry recordings were performed during light phase (ZT0-12).

### Transgenes and AAVs

All AAVs (serotype 1/2) were produced in house. The adenovirus helper plasmid *pFΔ6,* the AAV helper plasmids *pH21* (AAV1) and *pRVI* (AAV2), and the *pAAV t*ransgene plasmids were co-transfected into HEK293 cells and the resulting AAVs collected on heparin columns, as described previously (Klugmann et al., 2005; Yu et al., 2015). Plasmid *pAAV-iCre-2A-Venus* was provided by Thomas Kuner (Abraham et al., 2010). In this *pAAV-iCre-2A-Venus* plasmid, we replaced the original promoter with a small fragment of the mouse histidine decarboxylase (*hdc*) gene basal promoter divorced from cell-type selective enhancers, which we have found this promoter fragment gives strong pan-neuronal expression (promoter sequence and plasmid have been deposited at Addgene, *pAAV-iCre-2A-Venus:* plasmid number 182499, *pAAV-iCre-2A-mCherry:* plasmid number 182246). Plasmid *pAAV-GFP* was a gift from John T. Gray (Addgene plasmid 32396). To create plasmids *pAAV-hsyn-GCaMP6s* and the *pAAV-hsyn-flex-GCaMP6s* (Addgene plasmid 184284), the *GCaMP6s* reading frame from *pGP-CMV-GCaMP6s* (Addgene plasmid 40753, gift of Douglas Kim) (Chen et al., 2013) was mutated into *pAAV-flex-hM3D_q-_mCherry* (Krashes et al., 2011), either removing the *flex-hM3D_q_-mCherry* component or keeping both sets of *loxP* sites (*pAAV-flex* backbone), respectively. For knocking down GluN1 expression cell type-selectively, the pPRIME system (Stegmeier et al., 2005), cloned into AAV transgenes, was used to generate shRNAs – see section (“Generation of shRNAs to target GluN1”).

### Surgeries

All surgeries used adult male and female mice, 8-12 weeks old and were performed under deep general anesthesia with isoflurane (3% induction/ 2% maintenance) and under sterile conditions. Before starting the surgery, mice were injected subcutaneously (s.c.) with Buprenorphine (Vetergesic 0.3 mg/mL, 1:20 dilution in 0.9% sterile saline solution, final 0.1mg/kg) and Carprofen (Rimadyl 50mg/mL, 1:50 dilution in 0.9% sterile saline solution, final 5 mg/kg) and then placed in a stereotaxic frame. Mouse core temperature was constantly checked by rectal probe while respiration rate was regularly checked by eye.

For AAV injections, the virus was injected at a rate of 0.1 μL/min using Hamilton microliter #701 10 μL syringes and a stainless-steel needle (33-gauge, 15 mm long). LPO coordinates used for bilateral injection sites were relative to Bregma: AP:+0.40 mm, ML: -/+ 0.75 mm, DV was consecutive, injecting half volume at +5.20 mm and half at +5.15 mm. A total volume of 0.3 μL each side was injected. Control AHA coordinates used for bilateral injections sites were relative to Bregma: AP: -0.58 mm, ML: -/+ 0.65 mm, DV: +5.60 mm and +5.50 mm for consecutive injections.

For sleep recordings, EEG screw electrodes were chronically implanted on mice skull and EMG wire electrodes (AS634, Coorner Wire) were inserted in the neck extensor muscles. EEG screws were placed on the skull at (relative to Bregma): AP: -1.50 mm, ML: -1.50 mm; AP:-1.50 mm, ML: -2.00 mm; AP: +1.50 mm, ML: -2.00mm.

For fiber photometry, a monofiber (Ø 200 μm, 0.37 NA, Doric Lenses) was chronically implanted together with EEG and EMG electrodes. The fiber was positioned after AAV injections above the LPO following coordinates relative to Bregma: AP: +0.10 mm, ML: - 0.90 mm, DV: -5.00 mm.

For all surgeries, the wound was sewed around the head stage and the mouse was left recovering in a heat box. All instrumented mice were single housed to avoid lesions to the head stage. After surgery, mice injected with AAVs were allowed 1 month for recovering and for the viral transgenes to adequately express before being fitted with Neurologger 2A devices (see below) and undergoing any experimental procedures.

### EEG/EMG recordings and analysis

EEG and EMG traces were recorded using Neurologger 2A devices as described previously (Vyssotski et al., 2009; Anisimov et al., 2014; Gelegen et al., 2014), at a sampling rate of 200 Hz. The data obtained from the Neurologger 2A were downloaded and visualized using Spike2 Software (Cambridge Electronic Design, Cambridge, UK). The EEG was high pass filtered (0.5Hz, -3dB) using a digital filter, while EMG was band pass filtered between 5-45 Hz (-3dB). To define the vigilance states of Wake, NREM and REM sleep, delta power (0.5-4.5 Hz) and theta (5-10 Hz) power were calculated, as well as theta:delta power ratio and the EMG integral. Automated sleep scoring was performed using a Spike2 script and the result was manually corrected. For the three vigilance states, percentage amounts were calculate using costume Spike2 scripts. For sleep architecture analysis, costume MATLAB scripts were used. For stage transitions, we calculated each stage change reported in the hypnogram. The baseline number of transitions for animals lacking NMDA receptors has been represented as percentages over the control group. For sleep-deprived mice, we represented the transition numbers after 6hr SD as a percentage over the baseline value for each animal, in both control and experimental groups. Fast Fourier transformation (512 points) was used to calculate EEG power spectra.

### Sleep deprivation protocol and drug testing

Mice were fitted with Neurologger 2A devices and the 24h sleep-wake baseline (BL) was recorded. After BL, mice with Neurologger 2A devices were sleep deprived from ZT0 for 6 hours by introducing novel objects into their home cage (Tobler et al., 1997). To make the procedure minimally stressful, mice were never touched, apart from when changing cages. Sleep recordings were stopped at ZT24(0).

Dexmedetomidine injections were prepared from stock solution of 0.5 mg/mL (Dexdomitor), diluted in sterile saline before injections. Mice were injected (*i.p*.) with a dose of 25 or 50 μg/kg at ZT21 to record sleep and fragmentation phenotype. Zolpidem injections were prepared by dissolving zolpidem tartrate powder (Sigma-Aldrich) in sterile saline. Mice were injected (*i.p.*) with 5 mg/kg of solution at ZT23 and their EEG/EMG traces recorded for at least 24 hours.

### Histology and immunostaining

Animals were perfused transcardially with 20 mL of cold 1x PBS at a rate on 4 mL/min, followed by 20 mL 4% paraformaldehyde (PFA, 4 mL/min) in 1x PBS. Brains were dissected and post-fixed in 4% PFA overnight, and then transferred in 30% sucrose. After 3 days in sucrose, brains were cut in 35-μm coronal slice using a microtome (Leica). For staining, slices were transferred in an epitope retrieval solution (0.05% Tween-20, 10 mM sodium citrate buffer, and pH 6.0) for 20min at 82 °C, then left at room temperature (RT) for 15min before being washed. After 3 washes of 10min in 1x PBS, brain slices were blocked in 0.2% Triton™ x-100 (Sigma-Aldrich), 20% Normal Goat Serum (NGS, Vector Laboratories) in 1x PBS for 1h at RT, shaking. Primary antibody staining was then performed overnight at 4 °C shaking in 0.2% Triton, 2% NGS in 1x PBS. In case of double staining, both primary antibodies were added in the solution unless cross-reactivity was previously observed. The following day, slices were washed 3 times in 1x PBS for 10 minutes and then secondary antibody solution was applied for 1h and 30 min in 0.2% Triton, 2% NGS in 1x PBS, at RT shacking. If double staining was required, washes and another secondary antibody incubation were carried out. After secondary antibodies incubations, slices were washed again for 3 times for 10min, RT in 1x PBS shacking and DAPI staining (1:5000 in PBS, Hoechst 33342, Life Technologies) was then performed for a maximum of 10min. After at least 1 wash in 1x PBS, slices were ready to be mounted. For mounting, microscope slides (Superfrost PLUS, Thermo Scientific), mounting media ProLong™ Gold Antifade Reagent (Invitrogen) and glass cover slides (24 x 50 mm, VWR Internartional) were used. Primary antibodies: rabbit anti-GFP (Invitrogen, A6455, 1:1000), chicken anti-GFP (Abcam, ab13970, 1:1000), rat anti-mCherry (Invitrogen, M11217, 1:1000). Secondary antibodies (all from Invitrogen): Alexa Fluor-488 goat anti-Chicken (A11039, 1:500), Alexa Fluor-488 goat anti-rabbit (A11008, 1:500), Alexa Fluor-594 goat anti rabbit (A11072, 1:500) and Alexa Fluor-568 goat anti-rat (A11077, 1:500).

### Acute slice preparation and electrophysiology recordings

Mice were euthanized by cervical dislocation and subsequent decapitation. The brain was rapidly retrieved to be sliced and placed into cold oxygenated N-Methyl-D-glucamine (NMDG) solution (in mM: NMDG 93, HCl 93, KCl 2.5, NaH_2_PO_4_ 1.2, NaHCO_3_ 30, HEPES 20, glucose 25, sodium ascorbate 5, Thiourea 2, sodium pyruvate 3, MgSO_4_ 10, CaCl_2_ 0.5). Para-horizontal slices (thickness 300 μm) encompassing the LPO area were obtained using a vibrotome (Vibrating Microtome 7000smz-2; Campden Instruments LTD, UK). Slices were incubated for 15min in NMDG solution at 33 °C with constant oxygenation and transferred to oxygenated standard aCSF (in mM: NaCl 120, KCl 3.5, NaH_2_PO_4_ 1.25, NaHCO_3_ 25, glucose 10, MgCl_2_ 1, CaCl_2_ 2) solution for at least 1 hour at room temperature. Slices were transferred to a submersion recording chamber and were continuously perfused at a rate of 4-5ml/min with fully oxygenated aCSF at room temperature. For whole-cell recording, patch pipettes at 4-6 MΩ were pulled from borosilicate glass capillaries (1.5mm OD, 0.86 mm ID, Harvard Apparatus, #GC150F-10) and filled with intracellular solution containing in mM: 128 CsCH_3_SO_3_, 2.8 NaCl, 20 HEPES, 0.4 EGTA, 5 TEA-Cl, 2 Mg-ATP, 0.5 NaGTP (pH 7.35, osmolality 285 mOsm). 0.1% Neurobiotin was included in the intracellular solutions to identify the cell position and morphology following recording. Recordings were performed using a Multiclamp 700B amplifier (Molecular Devices. CA). Access and input resistances were monitored throughout the experiments. The access resistance was typically < 20 MΩ, and results we discarded if resistance changed by more than 20%.

GFP-positive neurons were visually identified and randomly selected. For AMPA and NMDA currents, a bipolar stimulus microelectrode (MX21AEW, FHC) was placed 100-200 μm away caudally from the recording site. The intensity of stimulus (10 ms) was adjusted to evoke a measurable, evoked EPSC in recording cells. AMPA and NMDA mixed currents were measured at a holding potential of +40 mV. After obtaining at least 10 sweeps of stable mixed currents, D-AP5 (50 μM) was perfused in the bath solution for 15 min and AMPA currents were measured. NMDA currents were obtained by subtracting AMPA currents from mixed currents off-line. The peak amplitude of both currents was used for AMDA/NMDA ratio analysis. For sEPSCs, GFP-positive LPO neurons were voltage clamped at -70 mV. A stable baseline recording was obtained for 5-10 min. Frequency, amplitude, rise & decay time constants of sEPSCs were analyzed off-line with the Mini Analysis (Synaptosoft). Frequency was obtained from 2 min of recording. All recordings were made in the presence of picrotoxin (100 μM).

For immunohistochemistry following electrophysiological recordings, brain slices were post-fixed in 4% PFA overnight at 4 °C. PFA was then washed away 3 times for 10 min in 1x PBS and slices were blocked and permeabilized in 20% NGS or 2% Bovine Serum Albumin (BSA) for 3 hours shaking. Primary anti-GFP antibody to trace viral distribution was diluted in 2% NGS, 0.5/0.7% TritonX in PBS overnight at 4 °C shaking. After 4 washes in 1x PBS for 10min each, secondary antibody was diluted in 2% NGS and 0.5% TritonX for 3 hours at RT and shaking. After washes and to track Neurobiotin-filled neurons recorded by electrophysiology, an Alexa594-conjugated streptavidin (Invitrogen) was diluted 1:500 in 1% NGS, 0.5% TritonX and slices were incubated for 2-3 hours at RT. Four washes of 15 min and subsequent DAPI incubation for 10 min were performed before slices were mounted on glass slides.

### Calcium Photometry

Following 4 weeks of recovery, mice were acclimatized to the testing environment for at least 2 hours before behavioral experiments and then recorded for 6 hours during the light period. The light source was a 473-nm diode-pumped solid state (DPSS) laser with fiber coupler (Shanghai Laser & Optics century Co.) and adjustable power supply (Shanghai Laser & Optics century Co.), controlled by a Grass SD9 stimulator. A lock-in amplifier (SR810, Stanford Research Systems, California, USA) was used to drive the laser at 125 Hz TTL pulses with an average power of 80 μW at the tip of the fiber directly connected to the mouse. The light source was coupled to a fluorescence cube (FMC_GFP_FC, Doric Lenses) through an optical fiber patch cord (Ø 200 μm, 0.22 NA, Doric Lenses). From the filter cube, an optical patch cord (Ø 200 μm, 0.37 NA, Doric Lenses) was connected to the monofiber chronically implanted in the mouse brain using ceramic sleeves (Thorlabs). The GCaMP6s output was then filtered at 500-550 nm through the fluorescence cube, converted to a voltage by a photodiode (APD-FC, Doric Lenses) and then amplified by the lock-in amplifier with a time constant of 30 ms. Finally, the signal was digitalized using a CED 1401 Micro box (Cambridge Electronic Design, Cambridge, UK) and recorded at 200 Hz using Spike2 software (Cambridge Electronic Design, Cambridge, UK). Photometry, EEG and EMG data were aligned offline using Spike2 software and analyzed using custom MATLAB (MathWorks) scripts. For each experiment, the photometry signal *F* was normalized to baseline using the function *ΔF/F = (F-F_0_)/F_0_*, where *F*_0_ is the mean fluorescence across the signal analyzed. When scatter plots of *ΔF/F* levels were plotted for each behavioral state, ΔF/F values were obtained by calculating the average F over a 50s rolling window, with a 0.2 Hz sampling rate over 70-90 minutes of photometry recordings. We applied a custom Matlab script to correct the baseline photometry values for photobleaching and photometry signal drift during long recordings.

### Generation of shRNAs to target GluN1

We used the Potent RNA Interference using MicroRNA Expression (PRIME) system, where shRNAs are placed into the context of a mir30 microRNA sequence (Stegmeier et al., 2005). By consulting the website http://katahdin.cshl.org, three shRNAs were designed to target exons 11 to 18 of the *Grin1* gene, encoding the region from amino acids 409 to 683 of GluN1. The sequences were amplified from mouse genomic DNA using primers (pSM2C Forward: 5'-GATGGCTG-CTCGAG-AAGGTATAT-TGCTGTTGACAGTGAGCG-3'; pSM2C Reverse: 5'-GTCTAGAG-GAATTC-CGAGGCAGTAGGCA-3'), following the protocol previously described (Stegmeier et al., 2005).

The three sequences were referred to as shRNA-GluN1-1.1, -2 or -3 (the underlined sequences are the 22-mers specific for the GluN1 subunit):

shRNA-GluN1.1 targeted the GluN1 sequence at 1800bp (600aa) of the coding sequence: 3’-

TGCTGTTGACAGTGAGCGAACTGACCCTGTCCTCTGCCATTAGTGAAGCCACAGATGTAATGGCAGAGGACAGGGTCAGTGTGCCTACTGCCTCGGA-5’

shRNA-GluN1.2 targeted the GluN1 sequence from 2565bp (855aa) of the coding sequence: 3’-

TGCTGTTGACAGTGAGCGCGCCGTGAACGTGTGGAGGAAGTAGTGAAGCCACAGATGTACTTCCTCCACACGTTCACGGCTTGCCTACTGCCTCGGA-5’

shRNA-GluN1.3 targeted the GluN1 sequence at 2215bp (738aa) of the coding region: 3’-

TGCTGTTGACAGTGAGCGCGGAGTTTGAGGCTTCACAGAATAGTGAAGCCACAGATGTATTCTGTGAAGCCTCAAACTCCATGCCTACTGCCTCGGA-5’

As a control for shRNA-GluN1 sequences, an shRNA scramble hairpin was used, not complementary to sequences in the mouse genome. The shRNA-scramble (scr) sequence was:

3’-GCTGTTGACAGTGAGCGAGCTCCCTGAATTGGAATCCTAGTGAAGCCACAGATGTAGGATTCCAATTCAGCGGGAGCCTGCCTACTGCCTCGGA-5’.

The three *shRNA-GluN1* hairpins and the *shRNA-scr* hairpin were cloned into the *pPRIME* vector to be then expressed and tested in HEK293 cells. To establish shRNA efficiencies in knocking down the NMDA GluN1 subunit expression, a plasmid was constructed expressing *GluN1-2A-mCherry* under the control of the CMV promoter. Each *GluN1-shRNA pPRIME* plasmid was then transfected into HEK293 cells together with *pGluN1-2A-mCherry.* After 60 hours in culture, mCherry fluorescence was quantified. The GluN1.3 shRNA produced lower fluorescence intensity, and thus higher inhibition of GluN1 expression, and it was therefore cloned into an AAV transgene cassette in an inverse orientation flanked by lox sites, as we described previously (Yu et al., 2015), to produce *pAAV-flex-GFP-shRNA-GluN1* with an *hdc* promoter fragment (this plasmid has been deposited at Addgene, number 182502) which was then packaged into AAV capsids (as above). The AAV transgene expresses GFP as well as shRNA-GluN1. For the controls, we packaged *pAAV-flex-GFP-shRNA-scramble* (this plasmid has been deposited at Addgene, number 182503).

### Experimental Design and Statistical Analysis

Origin, MATLAB and GraphPad Prism 8 were used for graphs and statistical analysis. Data collection and experimental procedure conditions were randomized. The experimenter was not blinded during the procedures. Data are presented as mean ± standard error of the means (SEM). The normality of each data set distribution was tested using the Kolmogorov-Smirnoff test. Paired/Unpaired two-tailed Student’s *t* test or one-way ANOVA were used to compare groups when only one variable was present. For longitudinal measurements, or measurements with two separate independent variables, a two-way repeated measures ANOVA, or a simple two-way ANOVA followed by *post-hoc* Sidak, Tukey, or Dunnett tests were performed. F and *p* values reported in the text are relative to the ANOVA statistical tests. Significantly different time points obtained through a multiple comparison analysis in the context of a two-way ANOVA test are indicated in the Figures, where *p < 0.05, ** *p* < 0.005, *** *p* < 0.0005, † *p* < 0.00005. When the data were not normally distributed, a Mann-Whitney test was performed. Statistical significance was considered when *p < 0.05, ** *p* < 0.005, *** *p* < 0.0005.

## Results

### Activity in LPO neurons is highest during REM sleep

**Figure 1:**
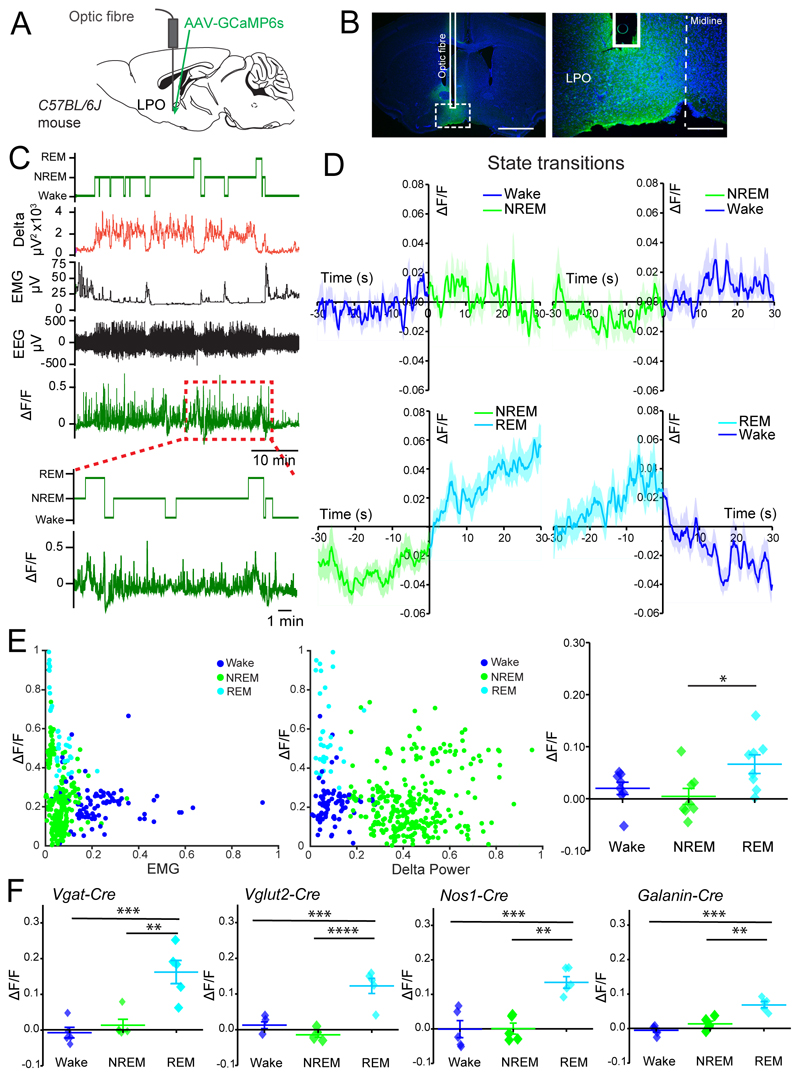
Activity in LPO hypothalamic neurons is highest during REM sleep. ***A***, schematic representation of optic fiber implantation and *AAV-GCaMP6s* injection in LPO area of *C57BL/6J* mice. ***B***, immunohistochemistry staining showing viral vector expression in LPO, using green fluorescent protein (GFP, in green) antisera and 4’,6-diamidino-2-phenylindole (DAPI, in blue). The optic fiber tract is also shown. Scale bars left 1 mm, right 200 μm. ***C***, example of photometry recording from LPO neurons aligned to EEG and EMG data. From the top: stage, delta power, EMG, EEG and *ΔF/F* from GCaMP6s signal corrected for baseline drift. The dotted red square indicates the segment of the trace expanded below. ***D***, photometry recordings across state transitions normalized as *ΔF/F* data points (means ± SEM). For the transitions to be considered, animals had to be in the behavioral state before and after transitions for at least 30s each. ***E***, left and center panels, scaled *ΔF/F* signal plotted against EMG (left) and delta power (right); right panel, quantification of LPO calcium activity as *ΔF/F* by behavioral state. ***F***, screening of LPO neuronal populations for calcium activity shown as *ΔF/F* using gene-specific Cre mouse lines (*Vgat-Cre, Vglut2-Cre, Nos1-Cre, Galanin-Cre*) and injection of *AAV-flex-GCaMP6s.* Data are mean ± SEM (*p < 0.05, **p < 0.005, ***p < 0.0005).

We first recorded neuronal activity in all LPO neurons using photometry with the calcium sensor GCaMP6s. Mice were injected in LPO with *AAV-GCaMP6s* (Fig. 1A-B). The highest calcium activity occurred during REM sleep episodes, especially at the beginning and end of the episodes (Figure 1C-D). During NREM sleep, LPO neurons showed a more sporadic and spiky activity, and during wakefulness only low activity. By plotting scaled means of the GCaMP6s signal against EMG signal (Fig. 1E, left panel) and delta power (Fig. 1E, center panel), REM sleep episodes were distributed towards higher values of GCaMP6s signal, separating them from the NREM sleep and Wake data points. Comparing the average of *ΔF/F* points during each behavioral state, REM sleep calcium values were significantly higher than in other vigilance states (one-way ANOVA: *F_(2,21)_* = 4.47, *p* =0.02; Tukey’s *post-hoc* test: Wake vs. NREM sleep *p* = 0.75, Wake vs. REM sleep *p* = 0.10, NREM vs. REM sleep *p* = 0.02, n = 8, Fig. 1E, right panel).

Photometry recordings were also performed in subtypes of LPO neurons by injecting *AAV-flex-GCaMP6s* into the LPO area of *Vgat-Cre, Vglut2-Cre, Nos1-Cre* and *Galanin-Cre* mice (Fig. 1F). As for the pan-neuronal recordings, these subsets all showed a significantly higher activity during REM sleep episodes compared with NREM sleep and Wake (*Vgat-Cre:* one-way ANOVA: *F_(2,12)_* = 16.87, *p* = 0.0003, Tukey’s *post-hoc* test: Wake vs. NREM sleep *p* = 0.79, Wake vs. REM sleep *p* = 0.0005, NREM vs. REM sleep *p* = 0.001, *n* = 5, *Vglut2-Cre:* one-way ANOVA: *F_(2,12)_* = 26.94, *p* < 0.0001, Tukey’s *post-hoc* test: Wake vs. NREM sleep *p* = 0.3, Wake vs. REM sleep *p* = 0.0003, NREM vs. REM sleep *p* <0.0001, *n* = 5, *NOS1-Cre:* one-way ANOVA: *F_(2,12)_* = 15.82, *p* = 0.0004, Tukey’s *post-hoc* test: Wake vs. NREM sleep *p* = 1.00, Wake vs. REM sleep *p* = 0.001, NREM vs. REM sleep *p* = 0.001, *n* = 5, *Galanin-Cre:* one-way ANOVA: *F_(2,12)_* = 21.55, *p* = 0.0001, Tukey’s *post-hoc* test: Wake vs. NREM sleep *p* = 0.28, Wake vs. REM sleep *p* = 0.0001, NREM vs. REM sleep *p* = 0.001, *n* = 5, Fig. 1F).

### Deletion of NMDA receptors from LPO neurons abolishes neuronal activity

**Figure 2:**
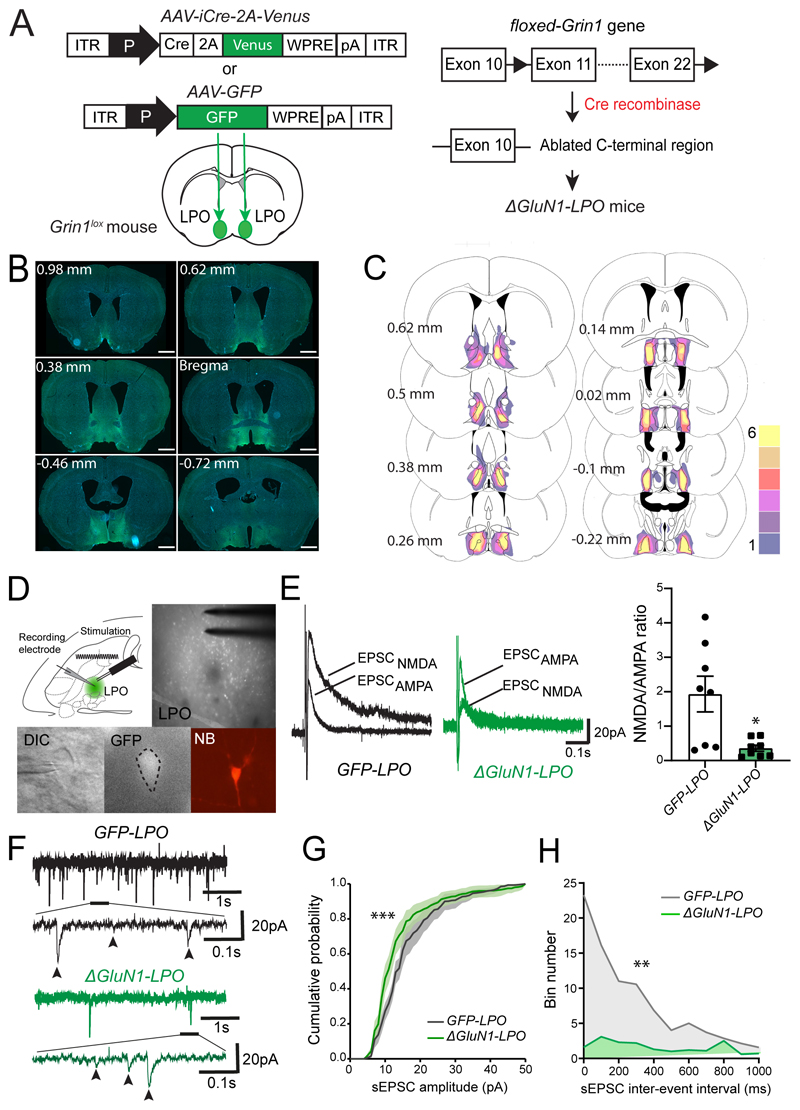
*AAV-Cre-Venus* mapping and characterization of excitatory post-synaptic currents (EPSCs) in *ΔGluN1-LPO* mice. ***A***, generation of *ΔGluN1-LPO* and *GFP-LPO* animals by bilateral injection in LPO of *AAV-Cre-2A-Venus* and *AAV-GFP* respectively. In *Grin1^lox^* mice, the c-terminal region of the *Grin1* gene is flanked by loxP sites (black arrowheads). When the Cre recombinase is expressed, it permanently excises the region between the loxP sites, and the *grin1* gene no longer encodes a functional NR1 subunit. Without the NR1 subunit, NMDA receptors cannot form. ***B***, mapping of Cre recombinase expression using immunohistochemistry (iCre-2A-Venus in green; DAPI in blue). Coordinates are relative to Bregma. Scale bars, 1 mm. ***C***, schematic representation of viral injection area and expression of the *AAV-Cre-2A-Venus* transgene *(ΔGluN1-LPO,* n = 6). In the heat map, yellow corresponds to areas were all 6 mice showed Venus expressing cells as detected by immunohistochemistry, whereas dark purple indicates areas where only 1 mouse showed Venus-positive cells. Coordinates are relative to Bregma. ***D**, top left,* schematic of para-horizontal *ex-vivo* brain slice showing the positioning of stimulus matrix and recording electrode. Top right, microscope images of GFP+ (Venus-positive) cells transduced with *AAV-iCre-2A-Venus* in LPO with stimulus electrode in sight. The cell successfully patched is shown in differential interface contrast (DIC), grey-scale for GFP and neurobiotin (NB) immune-detection (left to right, bottom row). ***E***, left panel, example traces of NMDA receptor- and AMPA receptor-mediated currents (labelled as EPSC_NMDA_ and EPSC_AMPA_ respectively) in *GFP-LPO* (in black) and *ΔGluN1-LPO* (in green) cells. Right panel, NMDA/AMPA ratios from *GFP-LPO* and *ΔGluN1-LPO* neurons showing a reduction of NMDA currents in *ΔGluN1-LPO* neurons. ***F***, Example of sEPSC recording from *GFP-LPO* (top, in grey) and *ΔGluN1-LPO* (bottom, in green) neurons. Part of each recording has been magnified to show the raw sEPSCs; currents are further indicated by arrowheads. ***G***, Cumulative probability histogram of sEPSCs amplitude. ***H***, cumulative bin number of inter-interval events (IEI) of sEPSCs. Data are mean ± SEM (*p < 0.05, **p < 0.005, ***p < 0.0005).

NMDA receptors assemble as heteromeric tetramers of subunits, with two core GluN1 subunits, whose gene, *grin1,* is transcribed throughout the brain (Moriyoshi et al., 1991), and GluN2 and/or GluN3 subunits, whose genes are differentially expressed (Monyer et al., 1992; Laurie and Seeburg, 1994; Monyer et al., 1994; Paoletti et al., 2013; Tovar et al., 2013). GluN1 is essential for all NMDA receptors (Tsien et al., 1996; Paoletti et al., 2013). Mice homozygous for a conditional allele (*Grin1^lox^*) that encodes the GluN1 subunit were bilaterally injected into the LPO with *AAV-iCre-2A-Venus,* generating *ΔGluN1-LPO* mice (Fig. 2A). Histological analysis confirmed that the *AAV-iCre-2A-Venus* transgene expression was restricted to the LPO area, with no spreading towards the medial preoptic or septal areas (n= 6, Figure 2B-C).

We examined if NMDA-receptor currents were deleted from *ΔGluN1-LPO* neurons compared with control *GFP-LPO* mice injected with *AAV-GFP* by recording evoked excitatory post-synaptic currents (eEPSCs) from *ex-vivo* acute slices prepared from the PO area (Fig. 2D-E). Both NMDA receptor-mediated slow currents (hundreds of milliseconds) and AMPA receptor-mediated fast currents (few milliseconds) were found on LPO neurons of *GFP-LPO* mice. NMDA receptor-mediated currents were smaller and the NMDA/AMPA ratio was significantly reduced in cells from *ΔGluN1-LPO* mice (two-tailed Mann Whitney test, U=10, *p* = 0.02. *ΔGluN1-LPO* (*n* = 8); *n*= 5, Fig 2E).

We also observed spontaneous EPSCs (sEPSCs, Fig 2F) and quantified their amplitude and frequencies in both experimental groups. As seen from the cumulative probability histogram of sEPSC amplitude, deletion of the NMDA receptor-mediated current from LPO neurons also reduced spontaneous excitatory post-synaptic current (sEPSCs) amplitudes (two-way ANOVA and Sidak’s *post-hoc* test: “probability”, *F_(50,765)_* = 130, *p* < 0.0001; “virus”: *F_(1, 765)_* = 25, *p* < 0.0001, Fig. 2G); and from determining the cumulative bin number of inter-interval events (IEI) of sEPSCs, the frequency of excitatory events was also reduced (two-way ANOVA and Sidak’s *post-hoc* test: “probability”, *F_(25, 390)_* = 3.1; “virus”: *F_(1, 390)_* = 15.3, *p* < 0.005, Fig. 2H).

**Figure 3:**
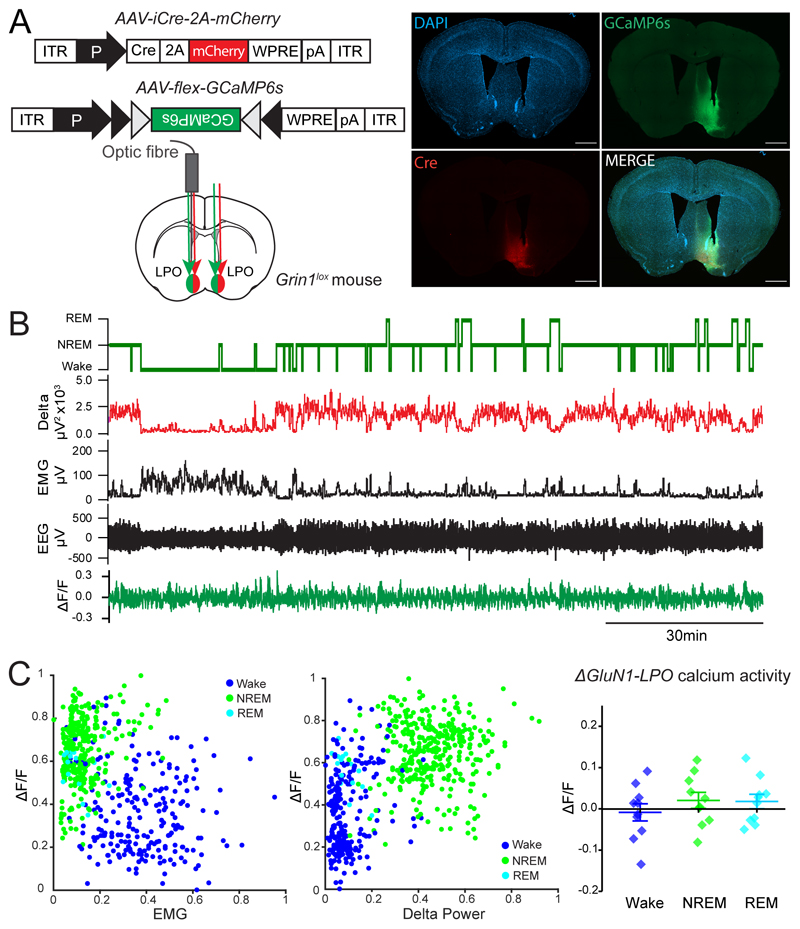
Activity in LPO hypothalamic neurons requires NMDA receptors. ***A**, Grin1^lox^* mice were bilaterally co-injected in LPO with *AAV-iCre-2A-mCherry* and *AAV-flex-GCaMP6s* to record calcium levels from neurons lacking NMDA receptors. An optic fiber was also implanted unilaterally for these recordings. On the right, immunohistochemistry showing from left to right: DAPI (blue) and *AAV-flex-GCaMP6s* transgene (green) on the first row, Cre recombinase (red) and merge on the second row. Scale bars represent 1 mm. ***B***, example of a photometry recording aligned to EEG and EMG in a *ΔGluN1-LPO* mouse. From the top: vigilance state, delta power, EMG, EEG and *ΔF/F* from GCaMP6s signal corrected for baseline drift. ***C***, left and center panels, scaled *ΔF/F* GCaMP6s signal plotted against EMG (left) and delta power (center); right panel, quantification of *ΔGluN1-LPO* calcium activity normalized as *ΔF/F* during behavioral states. Data in the right panel are mean ± SEM.

We next tested how NMDA receptor deletion from LPO neurons influenced intracellular calcium transitions. We co-injected into the LPO area of *Grin1^lox^* mice *AAV-iCre-2A-mCherry* and *AAV-flex-GCaMP6s,* so that only neurons expressing Cre recombinase expressed the calcium sensor (Fig. 3A). Regardless of vigilance state, calcium activity in *ΔGluN1-LPO* neurons was greatly reduced; indeed, no calcium fluctuations or variation from the baseline levels were observed during any of the 3 vigilance states in *ΔGluN1-LPO* neurons (one-way ANOVA: *F_(2,27)_* = 0.69, *p* = 0.51; Tukey’s *post-hoc* test: Wake vs. NREM sleep *p* = 0.54, Wake vs. REM sleep *p* = 0.60, NREM vs. REM sleep *p* = 0.99n = 5, Fig. 3B-C), and REM sleep was no longer highlighted by calcium activity, showing that deleting NMDA receptors ablates calcium fluctuations from LPO neurons.

### Deletion of NMDA receptors from LPO neurons reduces NREM and REM sleep and produces high sleep-wake fragmentation

**Figure 4:**
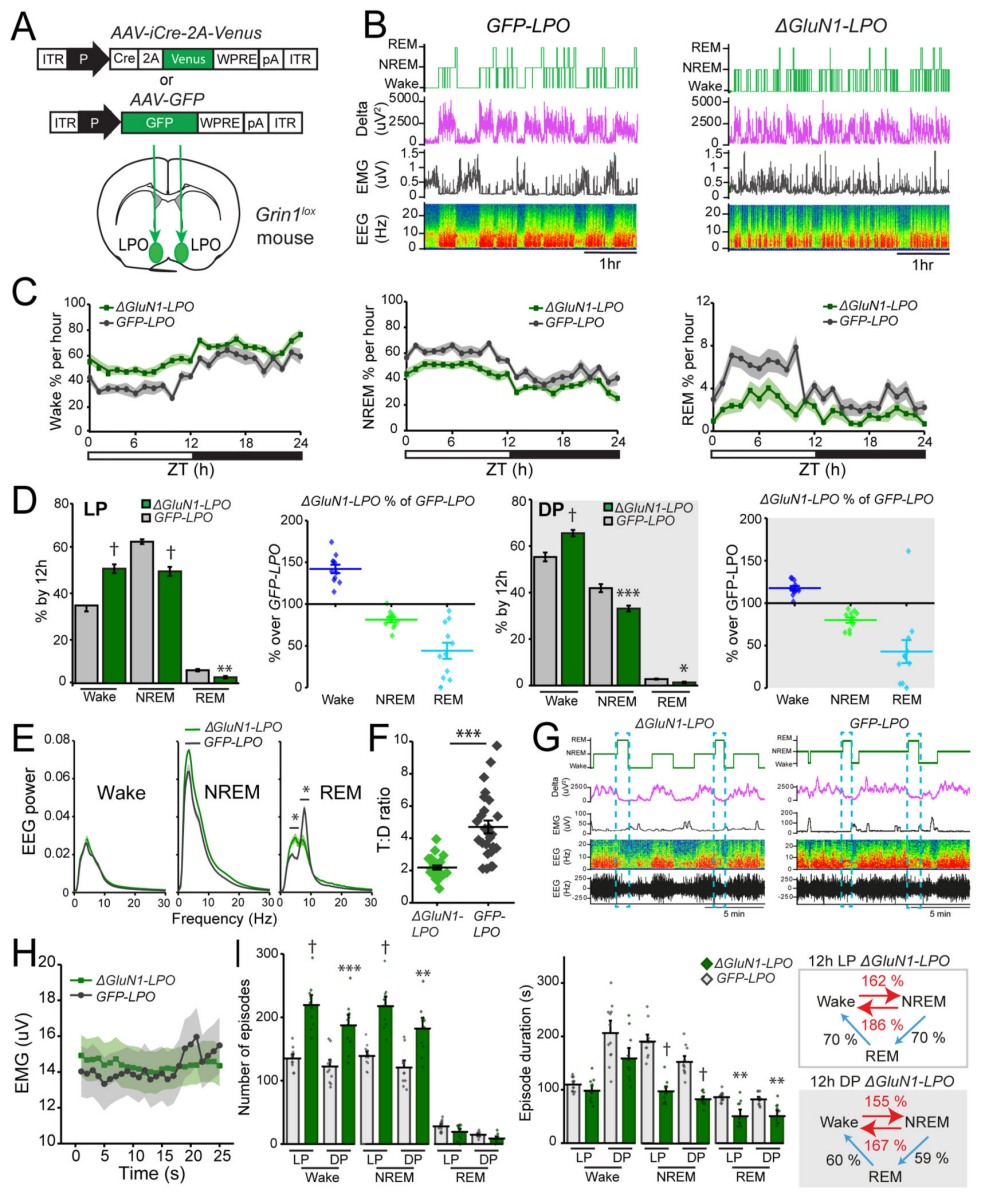
Deletion of NMDA receptors in LPO reduces NREM and REM sleep time and produces sleep-wake fragmentation. ***A**,* bilateral *AAV-iCre-2A-Venus* and *AAV-GFP* virus injections into LPO of *Grin1^lox^* mice to generate *ΔGluN1-LPO* and *GFP-LPO* control mice respectively. ***B***, example baseline recordings from *GFP-LPO* (left) and *ΔGluN1-LPO* (right) mice. From the top, hypnogram, delta power, EMG, and EEG represented as a somnogram and as a trace. ***C***, 24-hour baseline states distribution as percentage of 1h of wake (left), NREM (center) and REM sleep (right) during light period (LP: ZT0-12) and dark period (DP: ZT12-24). ***D***, 1^st^ and 3^rd^ panels, quantification of each behavioral state as percentage over LP (1^st^ panel) and DP (3^rd^ panel). 2^nd^ and 4^th^ panels, Wake, NREM and REM sleep amounts in *ΔGluN1-LPO* mice represented as percentage of *GFP-LPO* mice amounts during LP (2^nd^ panel) and DP (4^th^ panel). ***E***, EEG power spectrum for Wake (left), NREM (center) and REM sleep (right) normalized over total EEG power. ***F***, quantification of the theta: delta (T:D) ratio in *ΔGluN1-LPO* and *GFP-LPO* mice. Each point represents a 100s average of the T:D ratio during 3 REM episodes per animal. ***G***, Example baseline recordings showing EMG signal (atonia) during REM sleep bouts in both *ΔGluN1-LPO* (left) and *GFP-LPO* (right) mice. From the top, hypnogram, delta power, EMG, and EEG represented as a somnogram and as a raw trace. ***H***, EMG intensity during REM sleep episodes over 25s time. Values were exported at a 1Hz sampling rate from raw EMG traces. ***I***, left panel, episode number for 24h BL recordings assigned to LP and DP and behavioral state. Centre panel, episode mean duration for each behavioral state during LP and DP. Right panel, *ΔGluN1-LPO* mice vigilance state transitions during light (top) and dark (bottom) period represented as percentage over *GFP-LPO* transitions. In *C*, *D*, *E*, *F* and *I,* data are mean ± SEM (*p < 0.05, ** *p* < 0.005, *** *p* < 0.0005, † *p* < 0.00005).

We examined the effect on vigilance states of deleting GluN1 from LPO neurons (Fig. 4A-B). Over 24h, *ΔGluN1-LPO* mice compared with *GFP-LPO* mice spent more time awake (two-way repeated measures ANOVA and Sidak’s *post-hoc* test: Wake time, *F_(8.6, 181)_* = 14, *p* < 0.0001; NREM sleep time, *F_(9, 188)_* = 12.7, *p* < 0.0001; virus type, *F_(1, 21)_* = 41, *p* < 0.0001; REM sleep time: *F_(9, 190)_* = 8.3, *p* < 0.0001; virus type, *F_(1, 21)_* = 18, *p* = 0.0004; *ΔGluN1-LPO, n* = 11; *GFP-LPO, n* = 12; Fig. 4C). *ΔGluN1-LPO* mice lost on average 15-20% of NREM sleep time and 50% of their REM sleep time during both light and dark periods when compared to controls (2-way ANOVA and Sidak’s *post-hoc* test: light phase, vigilance state, *F_(2, 63)_* = 795, *p* = 0.0001; virus type, *F_(1, 63)_* = 0.0003, *p* = 0.98; dark phase, vigilance state, *F_(2, 63)_* = 957, *p* < 0.0001; virus type, *F_(1, 63)_* = 0.00001, *p* = 1; *ΔGluN1-LPO, n* = 11; *GFP-LPO, n* = 12; Fig. 4D). EEG power spectra for Wake and NREM sleep were similar between control and *ΔGluN1-LPO* mice (two-way ANOVA and Sidak’s *post-hoc* test: Wake frequency, *F_(89, 1800)_* = 746, p < 0.0001; virus type, *F_(1, 1800)_* = 9.1 x 10^-9^, *p* =1; NREM sleep frequency: *F_(89, 1800)_* = 1601, *p* < 0.0001; virus type, *F_(1, 1800)_* = 1.4 x 10^-8^, *p* = 1; REM sleep frequency, *F_(89, 1800)_* = 399, *p* < 0.0001; virus type, *F_(1, 1800)_* = 4.2 x 10^-9^, *p* = 1; *n* = 11; *GFP-LPO, n* = 12; Fig. 4E); however, *ΔGluN1-LPO* mice had strongly reduced cortical theta oscillations during REM sleep episodes (5-10 Hz, Figure 4E, right panel). Consequently, EEG and EMG recordings during REM sleep episodes showed a reduced theta:delta power (T:D) ratio in *ΔGluN1-LPO* mice compared with controls (unpaired two-tailed student’s *t* test, *t* = 6.076, df = 50, *p* < 0.0005; *GFP-LPO,* n=7; *ΔGluN1-LPO,* n=7; Fig 4F), although muscle atonia, the other hallmark of REM sleep, was maintained in both groups (Fig 4G). To verify this, we plotted raw EMG values during REM sleep episodes, finding no differences between the experimental groups (two-way ANOVA and Sidak’s *post-hoc* test: interaction, *F_(24, 1200)_* = 0.15, *p* > 0.99; time, *F_(24, 1200)_* = 0.18, *p* > 0.99; group, *F_(1, 1200)_* = 0.15, *p* = 0.7. *ΔGluN1-LPO, n* = 5; *GFP-LPO, n* = 5; 5 episodes per animal. Fig. 4H).

In addition to sleep loss and reduced cortical theta power, *ΔGluN1-LPO* mice had a highly fragmented sleep-wake phenotype: they lacked long Wake and NREM sleep episodes, as they had significantly more Wake and NREM sleep episodes (two-way ANOVA and Sidak’s *post-hoc* test: vigilance state, *F_(5, 126)_* = 232, *p* < 0.0001; virus type, *F_(1, 126)_* = 122, *p* < 0.0001; *ΔGluN1-LPO*, *n* = 11; *GFP-LPO*, *n* = 12 mice; Fig. 4I, left panel), with a decrease in their mean duration (two-way ANOVA and Sidak’s *post-hoc* test: vigilance state, *F_(5, 126)_* = 60.36, *p* < 0.0001;virus type, *F_(1, 126)_* = 105, *p* = 0.0001; *ΔGluN1-LPO, n* = 11; *GFP-LPO, n* = 12; Fig. 4I, center panel). Indeed, removing the NMDA receptor from LPO neurons increased transitions between wake to NREM sleep by > 55% and NREM sleep to wake by >80% during both light and dark period (unpaired two-tailed student’s *t* test, for the light phase: Wake → NREM, *ΔGluN1-LPO* = 213 ± 10, *GFP-LPO* = 131 ± 4 transitions, *t* = 7.410, df = 21, *p* < 0.0005; NREM Wake, *ΔGluN1-LPO* = 194 ± 10, *GFP-LPO* = 105 ± 6 transitions, *t* = 7.346, df = 21, *p*< 0.0005; for dark phase: Wake NREM, *ΔGluN1-LPO* = 179 ± 11, *GFP-LPO* = 116 ± 7 transitions, *t* = 4.839, df = 21, *p* < 0.0005; NREM → Wake, *ΔGluN1-LPO* = 171 ± 11, *GFP-LPO* = 102 ± 7 transitions, *t* = 5.261, df = 21, *p* < 0.0005, Fig. 4I, right panel). For REM sleep in *ΔGluN1-LPO* mice, there was a decrease in episodes number, in episodes durations and in transitions to and from this vigilance state (unpaired two-tailed Student’s *t* test, for the light phase, NREM→ REM and REM → Wake, *ΔGluN1-LPO* = 19 ± 3, *GFP-LPO* = 27 ± 2 transitions, *t* = 1.961, df = 21, *p* = 0.06; for the dark phase, NREM→ REM and REM → Wake, *ΔGluN1-LPO* = 8 ± 2, *GFP-LPO* = 14 ± 1 transitions, *t* = 2.741, df = 21, *p* = 0.012, Fig. 4I).

### Hypothalamic region-specific effect of NMDA receptor ablation on sleep-wake fragmentation

**Figure 5:**
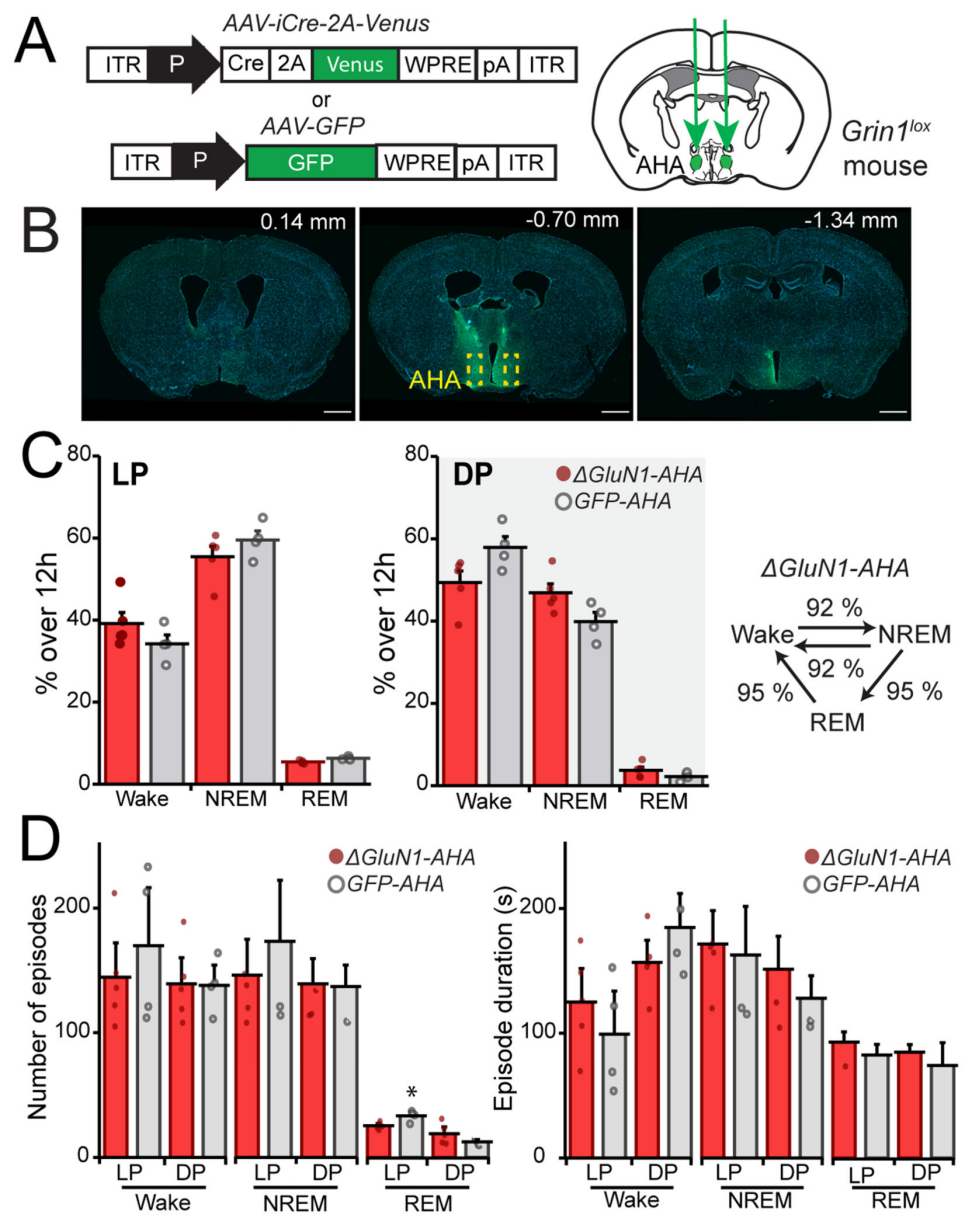
Deletion of the NR1 NMDA receptor subunit in the anterior hypothalamic area does not alter sleep-wake states. ***A***, *AAV-iCre-2A-Venus* or *AAV-GFP* bilateral injection in the anterior hypothalamic area (AHA), to generate *ΔGluN1-AHA* and *GFP-AHA* mice. ***B***, viral distribution from LPO to mid-thalamus – the AHA is indicated by the dashed yellow squares. Scale bars, 1 mm. ***C***, baseline state distribution shown as a percentage over 12h during light phase (LP) left panel, and dark phase (DP) center panel, comparing *ΔGluN1-AHA* with *GFP-AHA* mice, *right panel shows ΔGluN1-AHA* number of transitions between states over 24h represented as a percentage over the control *GFP-AHA* mice. ***D***, episode number (left) and mean duration (right) allocated by vigilance state and by light and dark period. In C and E data are means ± SEM.

As a control, we tested if deleting NMDA receptors in a region neighboring the PO area, the anterior hypothalamic area (AHA), caused sleep loss or fragmentation. Bilateral injection *of AAV-iCre-2A-Venus* and *AAV-GFP* into the AHA of *Grin1^lox^* mice, to generate *ΔGluN1-AHA* and *GFP-AHA* mice respectively (Fig. 5A-B) did not affect sleep and wake amounts during either the light or dark phases (two-way ANOVA and *post-hoc* Sidak’s test: light phase, virus type, *F_(1, 21)_* = 5.4 x 10^-6^, *p* = 1; vigilance state, *F_(2, 21)_* = 321, *p* < 0.0001. During dark phase, virus type, *F_(1, 21)_* = 1.9 x 10^-6^, *p* = 1; vigilance state, *F_(2, 21)_* = 328, *p* < 0.0001, Fig. 5C left and center panels). Nor did the deletion influence the number of transitions between vigilance states (unpaired two-tailed student’s *t* test, NREM → Wake, *ΔGluN1-AHA* = 232 ± 34, *GFP-AHA* = 254 ± 42 transitions, *t* = 0.4110, df = 7, *p* = 0.7; Wake → NREM, *ΔGluN1-AHA* = 274 ± 32, *GFP-AHA* = 299 ± 41 transitions, *t* = 0.4757, df = 7, *p* = 0.6; NREM → REM and REM → Wake, *ΔGluN1-AHA* = 43 ± 4, *GFP-AHA* = 45 ± 2 transitions, *t* = 0.4175, df = 7, *p* = 0.7; Fig. 5C right panel). *ΔGluN1-AHA* mice did not show sleep fragmentation: the episode number of NREM and REM sleep epochs and their mean duration were similar to *GFP-AHA* mice (two-way ANOVA and *post-hoc* Sidak’s test left panel, during light phase, virus type, *F_(1, 21)_* = 1.4, *p* = 0.2; vigilance state, *F_(2, 21)_* = 27, *p* < 0.0001; During dark phase, virus type, *F_(1, 21)_* = 0.1, *p* = 0.1; vigilance state, F_(2, 21)_ = 86, *p* < 0.0001; right panel during light phase, virus type *F_(1, 21)_* = 1, *p* = 0.3; vigilance state, *F_(2, 21)_* = 11, *p* < 0.0001; during the dark phase, virus type, *F_(1, 21)_* = 0.03, *p* = 0.9; vigilance state, *F_(2, 21)_* = 24, *p* < 0.0001; *GFP-AHA, n*= 4, *ΔGluN1-AHA, n* = 5. Fig. 5D). Therefore, the fragmented sleep phenotype produced by deleting NMDA receptors originates region-selectively in the preoptic hypothalamus.

### The insomnia of mice with NMDA receptors deleted from the LPO hypothalamus persists under high sleep pressure

**Figure 6:**
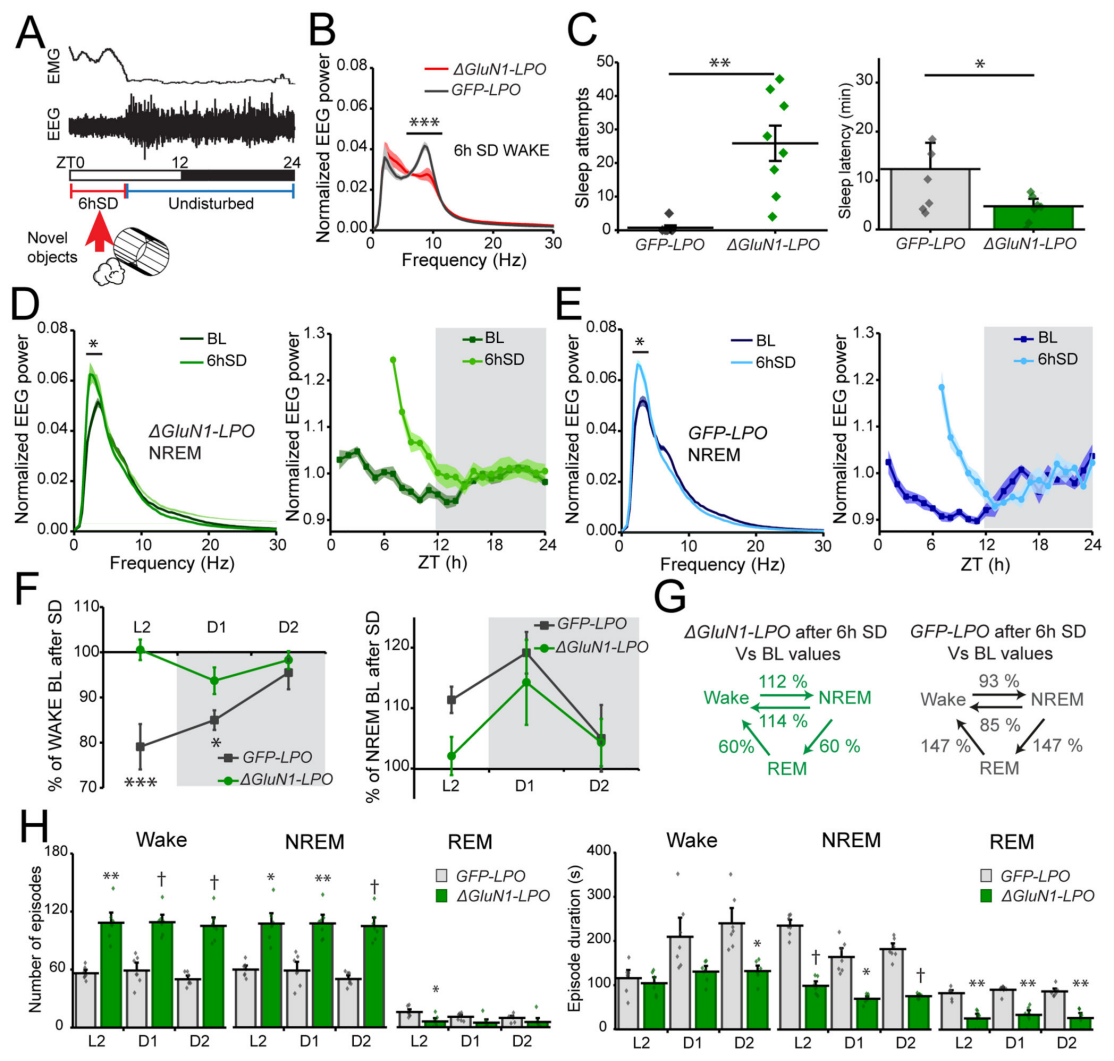
Deletion of the NMDA receptor from LPO does not affect sleep homeostasis and sleep-wake fragmentation persists in the recovery sleep following sleep deprivation (SD_. ***A***, representation of 6 h SD protocol starting when lights turn ON (ZT0), using novel objects to keep the animals awake. For the remaining 18h animals are left undisturbed. ***B***, Wake EEG power spectrum during 6h SD. ***C***, number of sleep attempts during the 6h SD (left) and latency to fall asleep (right) after SD, considering the first NREM sleep bout as at least 30s long. ***D*** and ***E***, left panels, NREM sleep EEG power spectrum during 1h following 6 h SD compared with same circadian time during baseline recordings in *ΔGluN1-LPO* (D) and in *GFP-LPO* (E) mice; right panels, NREM sleep EEG delta power calculated for every hour during baseline recordings and after 6h SD in *ΔGluN1-LPO* and *GFP-LPO* animals. The EEG power was normalized over the total power during each hour. F, percentage of Wake (left) and NREM sleep amounts (right) in *ΔGluN1-LPO* and *GFP-LPO* mice over their own baseline after 6 h SD. ZT6-12 (L2), ZT12-18 (D1), ZT18-24 (D2). ***G***, sum of state transitions in the 18h following 6h SD presented as a percentage over baseline for both *ΔGluN1-LPO* and *GFP-LPO* mice during the same circadian time. ***H***, left panel, episode number calculated over every 6 h following SD for Wake (left), NREM (center) and REM sleep (right). Right panels, episode mean duration calculated by 6 h following SD for Wake (left), NREM (center) and REM sleep (right). *GFP-LPO, n*=7; *ΔGluN1-LPO,* n=7. Data in all panels B, C, D, E, F and H are represented as means ± SEM (*p < 0.05, ** *p* < 0.005, *** *p* < 0.0005, † *p* < 0.00005).

In both fruit flies and mice, calcium entry through NMDA-type ionotropic glutamate-gated receptors has been proposed to signal the sleep homeostatic process (Liu et al., 2016; Tatsuki et al., 2016; Raccuglia et al., 2019). To investigate if the fragmented sleep of *ΔGluN1-LPO* mice persisted under high sleep pressure and if NMDA receptors in LPO were required for sleep homeostasis, we performed 6h of sleep deprivation (SD) at the onset of the “lights on” period (ZT0) when the sleep drive is highest (Fig. 6A). Although *ΔGluN1-LPO* mice were awake and moving during the sleep deprivation, there were several indications that they were under high sleep pressure. During the sleep deprivation, the EEG theta power in *ΔGluN1-LPO* mice was greatly reduced (red trace in Fig. 6B when compared with *GFP-LPO* mice), and most of the power was concentrated in the delta frequency band (two-way ANOVA and Sidak’s *post-hoc* test: EEG frequency, *F_(89, 1080)_* = 125, *p* < 0.0001; virus type, *F_(1,1080)_* = 4.3 x 10^-10^, *p* = 1; Fig. 6B). Compared with *GFP-LPO* mice, *ΔGluN1-LPO* mice had many more sleep attempts during the sleep deprivation procedure (Mann-Whitney test, U=1, *p* = 0.0006; Fig. 6C, left panel), and at the end of the 6-hour procedure they had a shorter latency to fall asleep (unpaired two-tailed student’s *t* test, *t* = 2.171, df = 13, *p* < 0.05, Fig. 6C, right panel).

During the subsequent first hour of sleep following sleep deprivation, both *ΔGluN1-LPO* and *GFP-LPO* mice had a significant increase in NREM sleep delta power compared with their own baseline at the same circadian time (two-way ANOVA and Sidak’s *post-hoc* test: *ΔGluN1-LPO*, power, *F_(89, 1080)_* = 653, *p* < 0.0001; virus, *F_(1, 1080)_* = 8.9 x 10^-9^, *p* = 1; *GFP-LPO,* power, *F_(89, 1080)_* = 963, *p* < 0.0001; virus, *F_(1, 1080)_* = 7.9 x 10^-9^, *p* = 1; Fig. 6D-E, left panels), showing that, by this measure, sleep homeostasis was intact. The typical diurnal variation in EEG delta power over 24 hours seen in control mice was also still present in *ΔGluN1-LPO* mice (Fig. 6D and E right panels). However, *ΔGluN1-LPO* mice were incapable of recuperating the sleep lost during sleep deprivation (Fig. 6F left panel). Following sleep deprivation as a percentage over their own baselines, *GFP-LPO* mice as expected increased their time asleep, reducing time spent awake. *ΔGluN1-LPO* mice, however, did not (two-way ANOVA and Sidak’s *post-hoc* test: Wake time, *F_(1.6, 19)_* = 5.8, *p* = 0.02; virus, *F_(1, 12)_* = 10.7, *p* = 0.007; NREM sleep time, *F_(1.5, 17.7)_* = 3.2, *p* = 0.08; virus, *F_(1, 12)_* = 2.9, *p* = 0.1, Fig. 6F right panel).

After sleep deprivation, *ΔGluN1-LPO* animals maintained the highly fragmented sleep phenotype during their recovery sleep, as shown by the percentage of transitions over their own baseline levels. Transitions to and from NREM sleep and Wake were still increased during recovery sleep, and transitions towards REM sleep decreased (paired two-tailed student’s *t*-test, Wake → NREM, 6hSD = 333±16, BL = 303±18, *t* = 1.324, df = 6, *p* = 0.23; NREM → Wake, 6hSD = 317±18, BL = 285±19, *t* = 1.296, df = 6, *p* = 0.24; NREM → REM and REM → Wake, 6hSD = 16±6, BL = 18±5, *t* = 0.234, df = 6, *p* = 0.8, Fig 6G, left panel). On the other hand, *GFP-LPO* control mice had decreased transitions between Wake and NREM sleep when compared with their baseline levels, and had increased transitions towards REM sleep, a sign of efficient recovery sleep (paired two-tailed student’s t-test, Wake → NREM, 6hSD = 159±8, BL = 175±10, *t* = 1.202, df = 6, *p* = 0.27; NREM → Wake, *6hSD*= 124±8, BL = 150±12, *t* = 2.373, df = 6, *p* = 0.06; NREM → REM and REM → Wake, 6hSD = 35±4, BL = 25±3, *t* = 2.785, df = 6, *p* = 0.03, Fig 6G, right panel). Additionally, in *ΔGluN1-LPO* mice, NREM sleep and wake episode numbers were still increased, and mean duration decreased after SD (two-way repeated measures ANOVA and Sidak’s *post-hoc* test: Wake, virus, *F_(1, 12)_* = 80, *p* < 0.0001; time, *F_(1.8, 22)_* = 1.8, *p* = 0.2; NREM, virus, *F_(1, 12)_* = 64.8, *p* < 0.0001; time, *F_(1.9, 23.2)_* = 1.8, *p* = 0.2; REM virus, *F_(1, 12)_* = 6.25, *p* = 0.03; time, *F_(1.9, 23)_* = 6.7, *p* = 0.006, Fig. 6H, left panel), whereas REM sleep values were consistently lower than in *GFP-LPO* mice (two-way repeated measures ANOVA and Sidak’s *post-hoc* test: Wake, virus, *F_(1, 12)_* = 17.3, *p* = 0.001; time, *F(1.9, 22.6)* = 13, *p* = 0.0002; NREM, virus, *F_(1, 12)_* = 202.5, *p* < 0.0001; time, *F_(1.7, 20)_* = 27, *p* < 0.0001; REM, virus, *F_(1, 12)_* = 64, *p* < 0.0001; time, *F_(1.8, 22)_* = 2.7, *p* = 0.1; *GFP-LPO,* n=7; *ΔGluN1-LPO, n*=7, Fig. 6H, right panel). The persistence of fragmentation after SD in *ΔGluN1-LPO* mice is noteworthy, as under increased sleep pressure, quantified by the delta power rebound, sleep is deeper compared with baseline levels. These data suggest that *ΔGluN1-LPO* mice were sleepy but could not stay asleep.

### Sedatives and sleeping medication transiently improve sleep of mice with NMDA receptors deleted from the LPO hypothalamus

**Figure 7:**
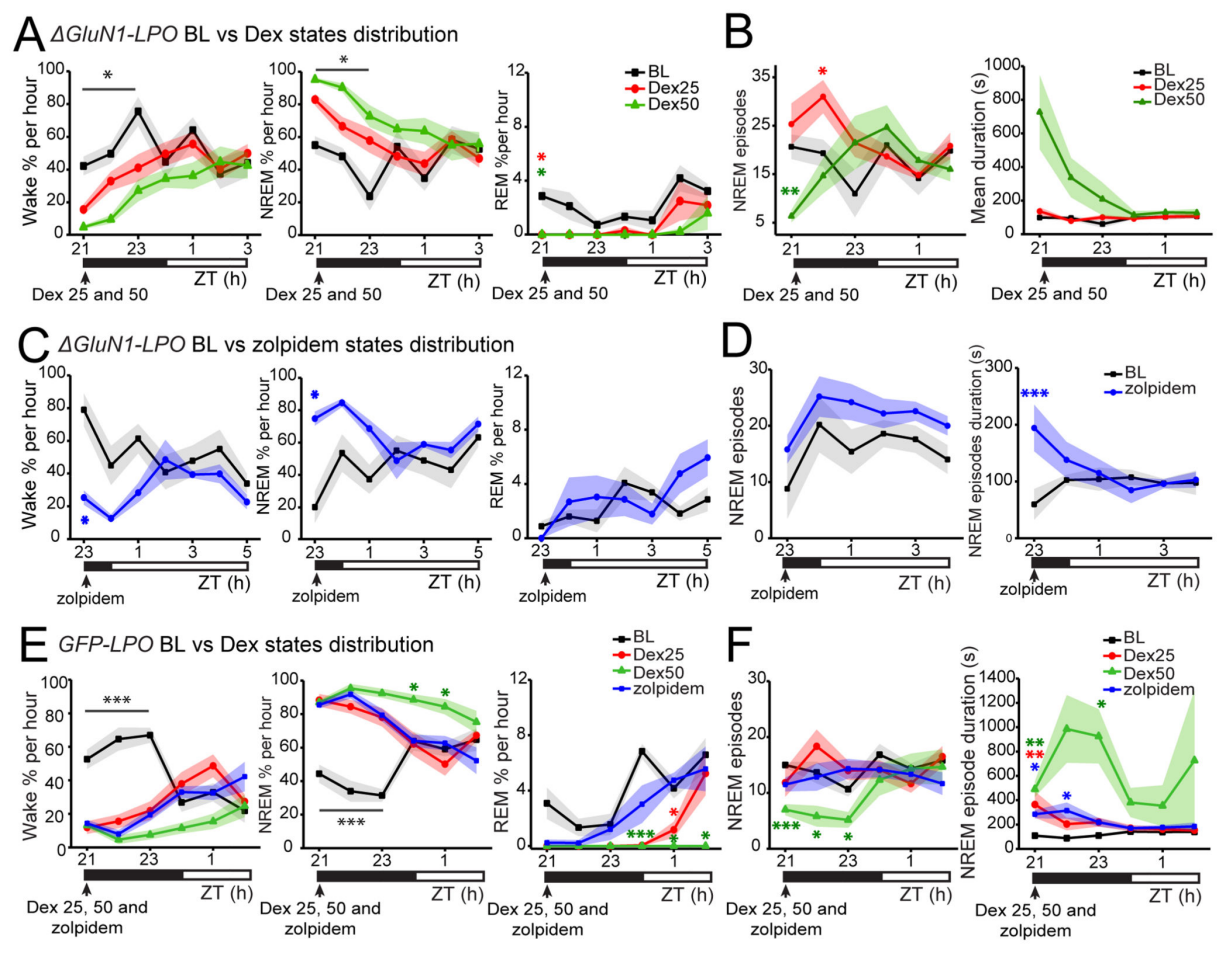
Dexmedetomidine and zolpidem both increased sleep time and dexmedetomidine reduced sleep fragmentation in mice lacking NMDA receptors in LPO. ***A***, *ΔGluN1-LPO* baseline (BL) states distribution as percentage of 1h in Wake (left), NREM (center) and REM sleep (right) compared with the distribution after *i.p.* injection of Dex 25 μg/kg (red) and 50 μg/kg (green) at ZT21. ***B***, *ΔGluN1-LPO* number of NREM sleep episodes (left) and NREM sleep episode mean duration (right) comparing BL to Dex sleep recordings, ***C***, *ΔGluN1-LPO* distribution by hour of Wake (left), NREM (center) and REM sleep (right) following *i.p.* injection of zolpidem (5 mg/kg) at ZT23. ***D***, NREM sleep episode number (left) and mean duration (right) comparing BL to zolpidem sleep recordings in *ΔGluN1-LPO* mice. ***E***, *GFP-LPO* BL state distribution as percentage of 1h in Wake (left), NREM (center) and REM sleep (right) compared with the distribution after injection at ZT21 of Dex 25 μg/kg (red), Dex 50 μg/kg (green) and zolpidem 5mg/kg (blue). ***F***, control *GFP-LPO* number of NREM sleep episodes (left) and NREM sleep episode mean duration (right) comparing BL to Dex and to zolpidem. For panels A, B, C and D, *ΔGluN1-LPO, n* = 6. For panels E and F, *GFP-LPO, n=* 6. Data in all panels are represented as mean ± SEM (*p < 0.05, ** *p* < 0.005, *** *p* < 0.005). Asterixis in black indicate significant difference from baseline values for each drug and dose tested. Asterixis in red, green, and blue indicated significant differences from BL values only for Dex 25μg/kg, Dex 50 μg/kg, and zolpidem respectively.

We investigated whether drugs that induce NREM-like sleep, dexmedetomidine (Dex) which is used in intensive care units for long-term sedation (Adams et al., 2013), and zolpidem (Ambien), a widely prescribed sleeping medication (Wisden et al., 2019), could reduce the high sleep fragmentation in *ΔGluN1-LPO* mice and restore consolidated sleep. Following *i.p.* injection, Dex (25 and 50 μg/kg) increased the time spent asleep (two-way repeated measures ANOVA and Tukey’s *post-hoc* test: for Wake time, time x dose, *F_(28, 210)_* = 2.4, *p* = 0.0002; *F(6.8, 102.5)* = 3.2, *p* = 0.004; dose, *F_(2, 15)_* = 8.1, *p* = 0.004; for NREM sleep time x dose, *F_(28, 210)_* = 2.8, *p* < 0.0001; time, *F_(6.8, 101.5)_* = 3.7, *p* = 0.002; dose, *F_(2, 15)_* = 7.6, *p* = 0.005; For REM sleep time x dose, *F_(28, 210)_* = 2.4, *p* = 0.0002; time, *F_(5.4, 80.9)_* = 13.7, *p* < 0.0001; dose, *F_(2, 15)_* = 1.8, *p* = 0.2, n = 6, Fig. 7A) and transiently changed, depending on the dose, the number of NREM sleep episodes (two-way repeated measures ANOVA and Tukey’s *post-hoc* test: time x dose, *F_(28, 210)_* = 2.87, *p* < 0.0001; time, *F_(6.2, 92.5)_* = 1.85, *p* = 0.1; dose, *F_(2, 15)_* = 1.7, *p* = 0.3; Fig. 7B, left panel) and increased NREM sleep episode mean duration for 1h after injection in *ΔGluN1-LPO* mice (two-way repeated measures ANOVA and Tukey’s *post-hoc* test: time x dose, *F_(28, 210)_* = 5.5, *p* < 0.0001; time, *F_(1.3, 19)_* = 5.7, *p* = 0.02; dose, *F_(2, 15)_* = 6.5, *p* = 0.009, Fig. 7B right panel). Similarly, zolpidem (5mg/kg) increased sleep time, and decreased wakefulness during the 6h post injection period (two-way repeated measures ANOVA and Tukey’s *post-hoc* test: for Wake time x drug, *F_(12, 96)_* = 3.61, *p* = 0.0002; time, *F_(4.3, 34.7)_* = 1.6, *p* = 0.2; dose, *F_(1, 8)_* = 6.4, *p* = 0.04; for NREM sleep time x drug, *F_(12, 96)_* = 3.98, *p* < 0.0001; time, *F_(4.4, 35)_* = 1.6 *p* = 0.2; dose, *F_(1, 8)_* = 6, *p* = 0.04; for REM sleep time x drug, *F_(12, 96)_* = 2.3, *p* = 0.01; time, *F_(4.3, 34.5)_* = 4.4, *p* = 0.005; dose, *F_(1, 8)_* = 2.2, *p* = 0.2, n = 6, Fig. 7C). However, zolpidem did not affect the number of NREM sleep episodes (two-way repeated measures ANOVA and Tukey’s *post-hoc* test: time x dose, *F_(12, 96)_* = 1.8, *p* = 0.6; time, *F_(5.5, 44)_* = 2, *p* = 0.07; dose, *F_(1, 8)_* = 0.7, *p* = 0.4, n = 6, Fig. 7D, left panel); but it increased their duration (two-way repeated measures ANOVA and Tukey’s *post-hoc* test: time x dose, *F_(12, 96)_* = 2.5, *p* = 0.007; time, *F_(12, 96)_* = 0.8, *p* = 0.7; dose, *F_(1, 8)_* = 3, *p* = 0.1, Fig. 7D, right panel). For both Dex and zolpidem, a few hours after drug administration the highly fragmented sleep pattern re-emerged (not shown).

Both doses of Dex (25 and 50 μg/kg), and zolpidem (5 mg/kg), had a similar effect on control *GFP-LPO* mice, increasing their NREM sleep time, while decreasing Wake and REM sleep times (two-way repeated measures ANOVA and Dunnett’s *post-hoc* test: for Wake time x drug, *F_(15, 100)_* = 12.44, *p* < 0.0001; time, *F_(3.2, 63.8)_* = 2.7, *p* = 0.5; dose, *F_(3, 20)_* = 19.7, *p* < 0.0001; for NREM sleep time x drug, *F_(15, 100)_* = 11.4, *p* < 0.0001; time, *F_(3.2, 65.2)_* = 6.4, *p* = 0.0005; dose, *F_(3, 20)_* = 28.9, *p* < 0.0001; for REM sleep time x drug, *F_(15, 100)_* = 3.8, *p* < 0.0001; time, *F_(2.5, 49.6)_* = 17.1, *p* < 0.0001; dose, *F_(3, 20)_* = 38, *p* < 0.0001, n = 6, Fig. 7E). Dex (50 μg/kg) and zolpidem - also decreased the number of NREM sleep episodes (two-way repeated measures ANOVA and Dunnett’s *post-hoc* test: time x drug, *F_(15, 100)_* = 2.3, *p* = 0.007; time, *F_(2.9, 59)_* = 2.8, *p* = 0.048; drug, *F_(3, 20)_* = 4.4, *p* =0.015, Fig. 7F, left panel) and increased NREM sleep average duration (two-way repeated measures ANOVA and Dunnett’s *post-hoc* test: time x drug, *F_(15, 100)_* = 1.02, *p* = 0.44; time, *F_(1.5, 30.8)_* = 1.2, *p* = 0.3; drug, *F_(3, 20)_* = 10.6, *p* =0.0002, Fig. 7F, right panel).

### Sleep fragmentation but not sleep loss is produced by selective NMDA GluN1 subunit knock-down in GABA LPO neurons but not glutamate LPO neurons

**Figure 8:**
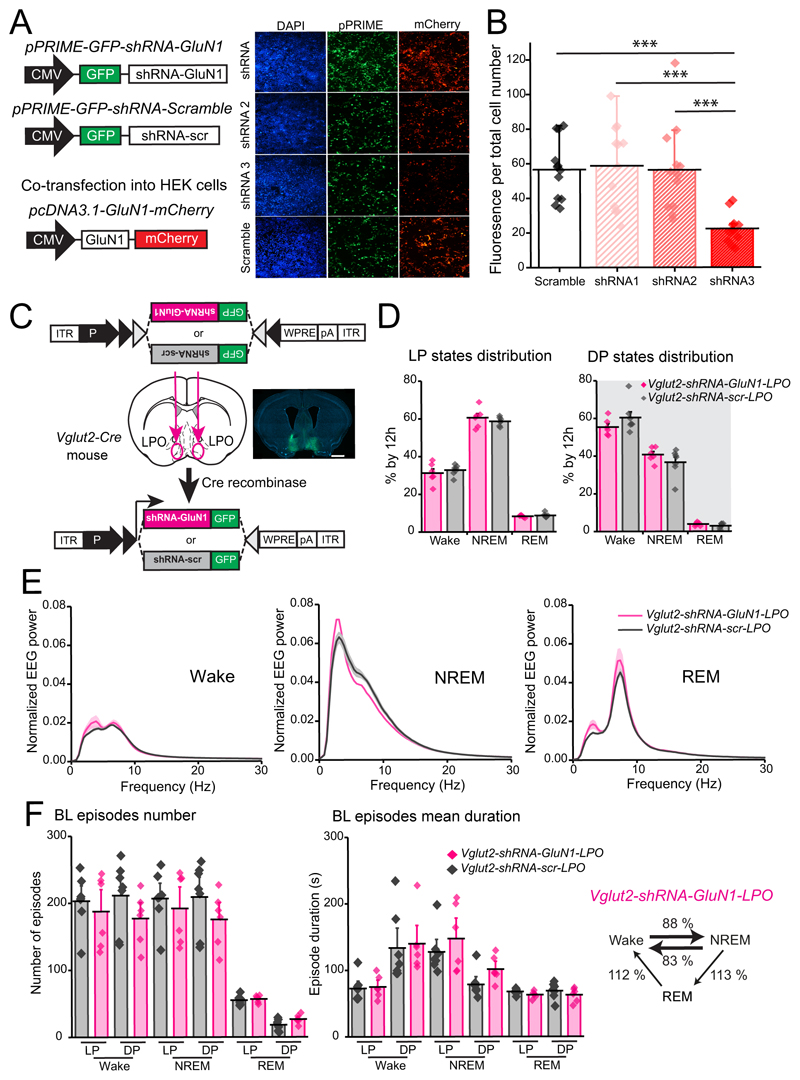
Knock-down of the NMDA receptor GluN1 subunit from *Vglut2*-expressing LPO neurons does not alter sleep and Wake patterns. ***A***, plasmid DNA constructs used to test *shRNA-GluN1* efficiency (left) and fluorescent images (right) of transfected HEK293 cells showing DAPI (left column, in blue), GFP reporter for expression of *shRNA-GluN1* and shRNA-scramble *pPRIME* vectors (central column, in green), and mCherry reporter expression, which indicates expression of the plasmid carrying the GluN1 sequence (right column, in red). ***B***, quantification of mCherry fluorescence per total cell number after transfection of HEK293 cells with plasmids expressing the shRNA-GluN1 or the control scramble shRNA). ***C***, *shRNA-GluN1* or *shRNA-scr* AAVs were bilaterally injected into the LPO of *Vglut2-Cre* mice. On the right, immunohistochemistry to map viral vector expression (GFP in green, DAPI in blue). Scale bar, 1 mm. ***D***, baseline (BL) vigilance state amounts calculated as a percentage over 12 h of the light (LP, left) and dark (DP, right) periods. ***E***, EEG power spectrum for Wake (left), NREM (center) and REM sleep (right) during 12h light period normalized over the total EEG power. ***F***, left panel, BL episode number over 12h comparing *Vglut2-shRNA-GluN1-LPO* and *Vglut2-shRNA-scr-LPO* mice. center panel, episodes mean duration by 12h during BL recordings; right panel, *Vglut2-shRNA-GluN1-LPO* number of transitions between states represented as percentage over the *Vglut2-shRNA-scr-LPO* BL. *Vglut2-shRNA-GluN1-LPO*, *n* = 6; *Vglut2-shRNA-scr-LPO, n* = 7. In B, C and D data are represented as mean ± SEM.

**Figure 9:**
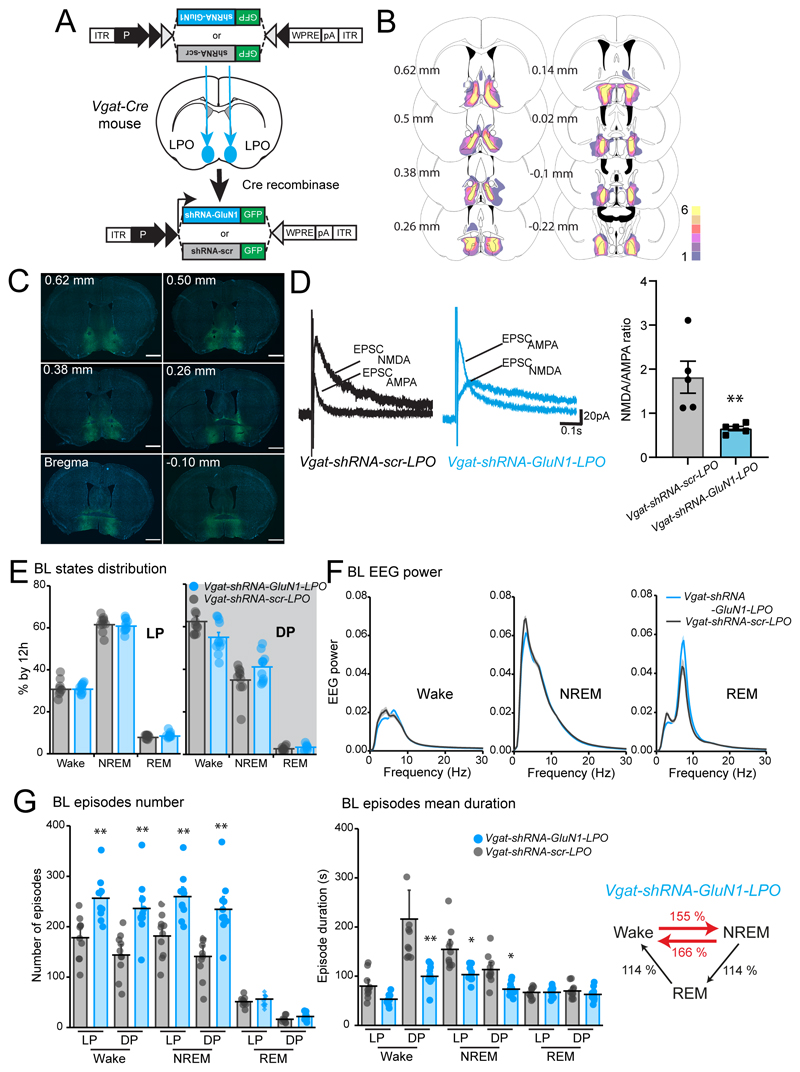
NMDA receptor GluN1 knock-down from GABA neurons in the LPO causes sleep-wake fragmentation but not sleep loss. ***A***, *AAV-flex-shRNA-GluN1* or *AAV-flex-shRNA-scramble* (scr) were bilaterally injected in the LPO area of *Vgat-cre* mice to generate *Vgat-shRNA-GluN1-LPO* and *Vgat-shRNA-scr-LPO* mice. ***B***, schematic representation of *AAV-flex-shRNA-GluN1* expression in the LPO of *Vgat-Cre* mice used for sleep recordings. In the heat map, yellow corresponds to areas were all 6 mice showed GFP-positive cells, whereas dark purple indicates areas where only 1 mouse showed GFP-positive cells. Coordinates are relative to Bregma. ***C***, mapping of shRNA transgene expression using immunohistochemistry (*shRNA-GluN1* expressing GFP in green; DAPI in blue). Coordinates are relative to Bregma. Scale bars, 1 mm. ***D***, left panel, example traces of NMDA receptor- and AMPA receptor-mediated currents (labelled as EPSC_NMDA_ and EPSC_AMPA_ respectively) in *Vgat-shRNA-scr-LPO* (in black) and *vgat-shRNA-GluN1-LPO* (in blue) cells. Right panel, NMDA/AMPA ratio in *Vgat-shRNA-scr-LPO* and *Vgat-shRNA-GluN1-LPO* neurons, showing a decrease in NMDA current in neurons expressing *shRNA-GluN1* compared with controls. ***E***, vigilance state distribution represented as a percentage of 12h during light (LP, left) and dark (DP, right) periods for *Vgat shRNA-GluN1-LPO* and *Vgat-shRNA-scr-LPO* mice. ***F***, EEG power spectrum of Wake (left), NREM (center) and REM (right) sleep during 12h light period in *Vgat-shRNA-GluN1* mice normalized over total EEG power. ***G***, left and center panels, baseline episode number (left) and episode mean duration (center) comparing *Vgat-shRNA-GluN1-LPO* with control *Vgat shRNA-scr-LPO* mice; right panel, number of transitions in *Vgat-shRNA-GluN1-LPO* mice represented as percentage over control group during 24h baseline recordings. *Vgat-shRN-GluN1-LPO, n* = 12; *Vgat-shRNA-scr-LPO, n* = 11. In B, D, E and F data are represented as mean ± SEM. In F, *p < 0.05, **p < 0.005.

To investigate the NR1-expressing LPO cell types involved in regulating sleep, we could not use Cre recombinase to ablate the *Grin1* gene because no cell type-selective promoters are available to restrict NR1 deletion selectively to subtypes of LPO cells without also affecting other brain areas. We therefore decided to use shRNA transgenes to reduce GluN1 expression cell-type selectively, for example, in GABAergic or glutamatergic cells in LPO (see Materials & Methods). First, we tested the efficacy of three different shRNAs to knockdown recombinant GluN1-mCherry cDNA expression (Fig. 8A and B). Having identified a suitable shRNA, shRNA3, that significantly reduced GluN1-mCherry cDNA expression compared with scrambled shRNA (one-way ANOVA: *F_(3, 44)_* = 9.13, *p* < 0.0001; Tukey’s *post-hoc* test: Scramble vs. shRNA3 *p* = 0.0007, shRNA1 vs. shRNA3 *p* = 0.0003, shRNA2 vs. shRNA3 *p* = 0.0008, n = 10 transfections, , Fig. 8B), the shRNA was cloned into an AAV transgene cassette (see Materials and Methods), and *AAV-flex-shRNA-GluN1* and *AAV-flex-shRNA-scramble* (*scr*)viruses were then bilaterally injected into the LPO areas of *Vglut2-Cre* and *Vgat-Cre* mice to generate *Vglut2-shRNA-GluN1-LPO* and *Vgat-shRNA-GluN1-LPO* mice and the associated scramble control respectively (Fig. 8C, Fig. 9A).

For the *Vglut2-shRNA-GluN1-LPO* mice, and unlike the *ΔGluN1-LPO* mice, the macrostructure of vigilance states was unchanged: *Vglut2-shRNA-GluN1-LPO* mice did not have sleep loss compared with scramble shRNA controls (two-way ANOVA and Sidak’s *post-hoc* test: For the light phase, vigilance state, *F_(2, 33)_* = 760, *p* < 0.0001; virus, F_(1, 33)_ = 2.8*10^-6^, *p* = 1. For the dark phase, vigilance state, *F_(2, 33)_* = 374, *p* < 0.0001; virus, *F_(1, 33)_* = 1.3 x 10^-7^, *p* = 1, Fig. 8D). The EEG power spectra during wake, NREM and REM sleep were also typical (Fig. 8E).

*Vglut2-shRNA-GluN1-LPO* mice did not have a sleep-wake fragmentation phenotype - the number of vigilance state episodes was unchanged compared with scramble shRNA controls (two-way ANOVA and Sidak’s *post-hoc* test: vigilance state, *F_(5,66)_* = 58, *p* < 0.0001; virus, *F_(1, 66)_* = 2.8, *p* = 0.1, Fig. 8F, left panel); the mean duration of vigilance states was the same as in mice injected with scramble shRNA controls (two-way ANOVA and Sidak’s *post-hoc* test: vigilance state, *F_5, 66_*) = 17.6, *p* < 0.0001; virus, *F_(1, 66)_* = 1, *p* = 0.3, Fig. 8F, centre panel); as was the number of transitions between states represented as percentage over number of transitions in *Vglut2-shRNA-scr-LPO* mice was also similar (unpaired two-tailed student’s *t* test, Wake → NREM, *Vglut2-shRNA-GluN1-LPO* = 356.5 ± 37, *Vglut2-shRNA-scr-LPO* = 405 ± 31 transitions, *t* = 1.009, df = 11, *p* = 0.3; NREM → Wake, *Vglut2-shRNA-GluN1-LPO* = 276 ± 34, *Vglut2-shRNA-scr-LPO* = 333 ± 31 transitions, *t* = 1.252, df = 11, *p* = 0.2; NREM → REM and REM → Wake, *Vglut2-shRNA - GluN1-LPO* = 81 ± 4, *Vglut2-shRNA-scr-LPO* = 73 ± 3 transitions, *t* = 1.629, df = 11, *p* = 0.1, Fig. 8F, right panel).

For the *Vgat-shRNA-GluN1-LPO* mice (Fig. 9A), we confirmed that the *AAV-flex-shRNA-GluN1* transgene expression was confined largely to LPO (Fig. 9B-C). We tested the efficiency of NMDA receptor knockdown by recording evoked EPSCs on Vgat-LPO neurons in acute hypothalamic slices. *Vgat-shRNA-GluN1-LPO* cells visibly showed a reduction in NMDA receptor-mediated currents compared with *Vgat-shRNA-scr-LPO* control cells and the NMDA/AMPA ratio was significantly reduced in *Vgat-shRNA-GluN1-LPO* cells (Fig 9D), demonstrating that *Vgat-LPO* neurons in *Vgat-shRNA-GluN1-LPO* mice have similar degree of NMDA receptor-mediated current deficiency as *ΛGluN1-LPO* neurons with the Cre-mediated disruption of the *grin1* gene (two-tailed Mann Whitney test, U=0, *p* = 0.008. *Vgat-shRNA-scr-LPO, n* = 5; *Vgat-shRNA-GluN1-LPO, n* = 5. Fig. 9D, right panel).

We next looked at the sleep phenotype of *Vgat-shRNA-GluN1-LPO* mice. The overall macrostructure of vigilance states was not changed: *Vgat-shRNA-GluN1-LPO* mice did not have sleep loss compared with the respective scramble shRNA controls (two-way ANOVA and Sidak’s *post-hoc* test: vigilance state, *F_(5, 113)_* = 564, *p* < 0.0001; virus, *F_(1, 113)_* = 0.03, *p* = 0.9, Fig. 9E). The EEG power spectra during Wake, NREM and REM sleep were also not different (Fig. 9F). However, for *Vgat-shRNA-GluN1-LPO* mice, there were more Wake and NREM sleep episodes (two-way ANOVA and Sidak’s *post-hoc* test: episode number, *F_(5, 114)_* = 110, *p* < 0.0001; virus, *F(1, 114)* = 73, *p* < 0.0001, Fig. 9G, left panel), and decreased in episode duration resembling the sleep fragmentation phenotype observed in *ΔGluN1-LPO* mice (two-way ANOVA and Sidak’s *post-hoc* test: mean duration, *F_(5, 114)_* = 39, *p* < 0.0001; virus, *F_(1, 114)_* = 62, *p* < 0.0001, Fig. 9G, centre panel). Additionally, there was a >55% increase in transitions between vigilance states in *Vgat-shRNA-GluN1-LPO* mice compared with *Vgat-shRNA-scr-LPO* mice (unpaired two-tailed student’s *t* test, Wake → NREM, *Vgat-shRNA-GluN1-LPO* = 484 ± 28, *Vgat-shRNA-scr-LPO* = 312 ± 25 transitions, *t* = 4.594, df = 19, *p* = 0.0002; NREM → Wake, *Vgat-shRNA-GluN1-LPO* = 408 ± 30, *Vgat-shRNA-scr-LPO* = 246 ± 26 transitions, *t* = 4.09, df = 19, *p* = 0.0006; NREM → REM and REM → Wake, *Vgat-shRNA-GluN1-LPO* = 76 ± 6 transitions, *Vgat-shRNA-scr-LPO* = 66 ± 3 transitions, *t* = 1.409, df = 19, *p* = 0.2. Fig. 9G, right panel).

**Figure 10:**
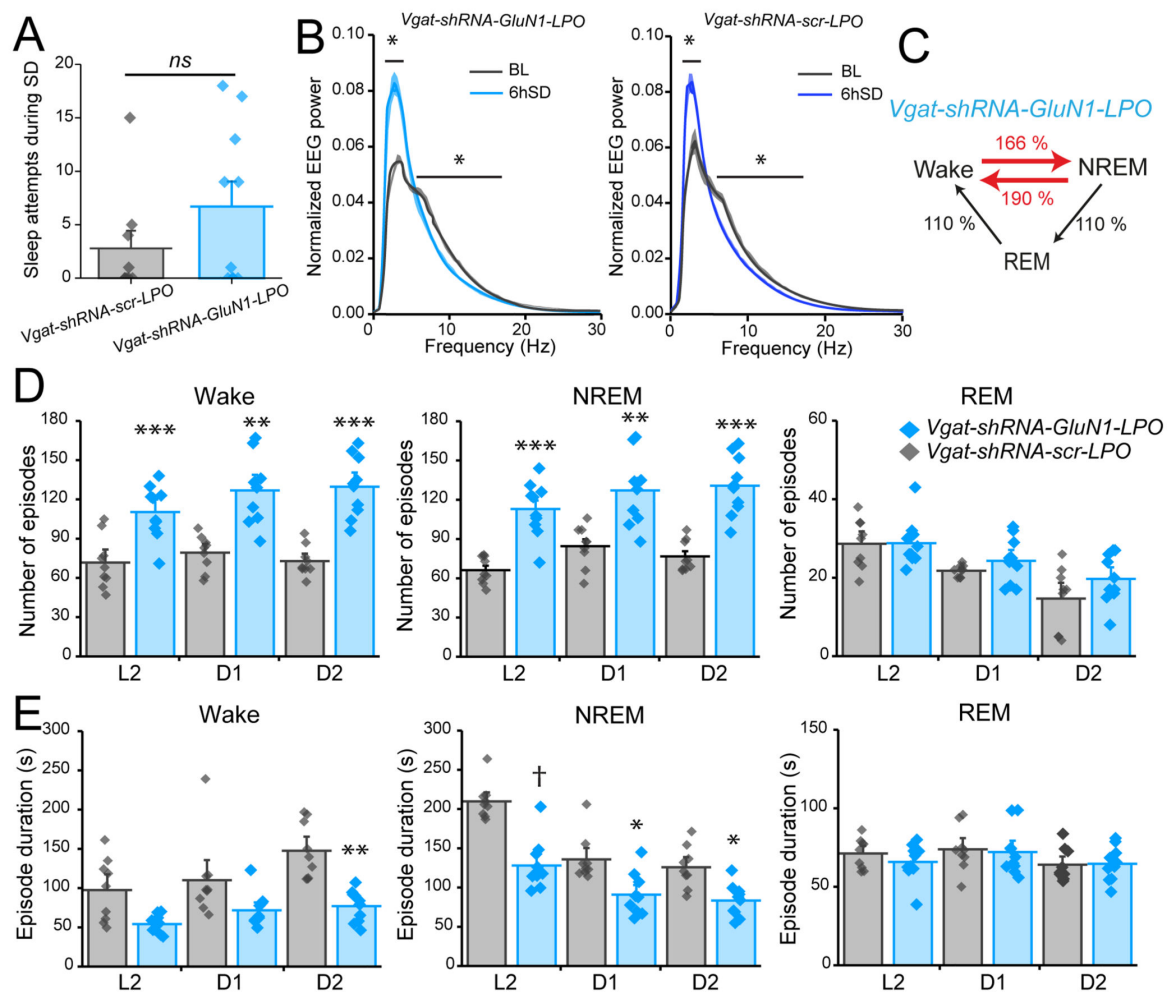
*Vgat-shRNA-GluN1-LPO* mice still have fragmented sleep after 6 h sleep deprivation. ***A***, number of NREM sleep attempts during 6 h sleep deprivation (SD) comparing *Vgat-shRNA-GluN1-LPO* with *Vgat-shRNA-scr-LPO* mice. ***B***, NREM EEG power spectrum comparing 1h after sleep deprivation to the same circadian time during baseline recordings, to represent EEG delta power rebound in *Vgat-shRNA-GluN1-LPO* (left) and *Vgat-shRNA-scr-LPO* (right) mice. ***C***, number of transitions for *Vgat-shRNA-GluN1-LPO* mice during the 18h following 6h SD represented as a percentage over the number of transitions exhibited by *Vgat-shRNA-scr-LPO* control mice. ***D***, episode number during Wake (left), NREM (centre) and REM sleep (right) following 6 h SD. ZT6-12 (L2), ZT12-18 (D1), ZT18-24 (D2). ***E***, episode mean duration following 6 h SD for Wake (left), NREM (centre) and REM sleep (right). *Vgat-shRNA-GluN1-LPO, n* = 10; *Vgat-shRNA-scr-LPO, n* = 9. Data in A, B, D and E are means ± SEM. In B, D and E, *p* < 0.05, ** *p* < 0.005, *** *p* < 0.0005, † *p* < 0.00005.

As for the *ΔGluN1-LPO* mice with the NMDA receptors removed from all cell types in the LPO area, we next tested if the sleep-wake fragmentation phenotype of *Vgat-shRNA-GluN1-LPO* mice persisted under conditions of raised sleep pressure following 6h of sleep deprivation. In contrast to mice with GluN1 deletion from all neuronal cell types in LPO, *Vgat-shRNA-GluN1-LPO* mice did not show a “sleepy phenotype”, as their sleep attempts during sleep deprivation did not differ from the control group (Kolmogorov-Smirnov test, *Vgat-shRNA-GluN1-LPO, n* = 10; *Vgat-shRNA-scr-LPO, n* = 9, *p* = 0.5, Fig. 10A). Following 6h SD, *Vgat-shRNA-GluN1-LPO* mice had a significant increase in the EEG delta power during the 1h following SD compared to their own baseline power, similarly to *ΔGluN1-LPO* mice, showing that sleep homeostasis was intact (2-way ANOVA and Sidak’s *post-hoc* test: for *Vgat-shRNA-GluN1-LPO* mice, frequency, *F_(89,1620)_* = 614, *p* < 0.0001; virus, *F_(1, 1620)_* = 1, *p* = 0.3; for *Vgat-shRNA-scr-LPO* mice, frequency, F_(89, 1620)_ = 624, *p* < 0.0001; virus, *F_(1, 1620)_* = 2, *p* = 0.1, *Vgat-shRNA-GluN1-LPO, n* = 10; mice; *Vgat-shRNA-scr-LPO, n* = 9 mice, Fig. 10B). *Vgat-shRNA-GluN1-LPO* mice maintained the fragmented sleep phenotype under high sleep pressure, with more transitions between states (unpaired two-tailed student’s *t* test, Wake → NREM, *Vgat-shRNA-GluN1-LPO* = 368 ± 19.4, *Vgat-shRNA-scr-LPO* = 221 ± 10.5 transitions, *t* = 6.43, df = 17, *p* < 0.0005; NREM → Wake, *Vgat-shRNA-GluN1-LPO* = 297 ± 18, *Vgat-shRNA-scr-LPO* = 156 ± 10, *t* = 6.6, df = 17, *p* < 0.0005; NREM → REM and REM → Wake, *Vgat-shRNA-GluN1-LPO* = 71 ± 4, *Vgat-shRNA-scr-LPO* = 64 ± 4 transitions, *t* = 1.097, df = 17, p= 0.3, Fig. 10C), and more wake and NREM episodes (two-way repeated measures ANOVA and Sidak’s *post-hoc* test: vigilance state, *F_(2.5, 43)_* = 218, *p* < 0.0001; virus, *F_(1, 17)_* = 37, *p* < 0.0001, Fig. 10D), and with decreased mean durations compared with *Vgat-shRNA-scr-LPO* mice (two-way repeated measures ANOVA and Sidak’s *post-hoc* test: vigilance state, *F_(4.8, 81)_* = 56, *p* < 0.0001; virus, *F_(1, 17)_* = 40, *p* < 0.0001). *Vgat-shRNA-GluN1-LPO, n* = 10; *Vgat-shRNA-scr-LPO, n* = 9 mice, Fig. 10E). Thus, the sleep-wake fragmentation aspect but not the REM sleep loss of *ΔGluN1-LPO* mice originates from GABA cells in LPO.

### Sedatives and sleeping medications improve sleep in mice lacking NMDA receptors in LPO GABA neurons

**Figure 11:**
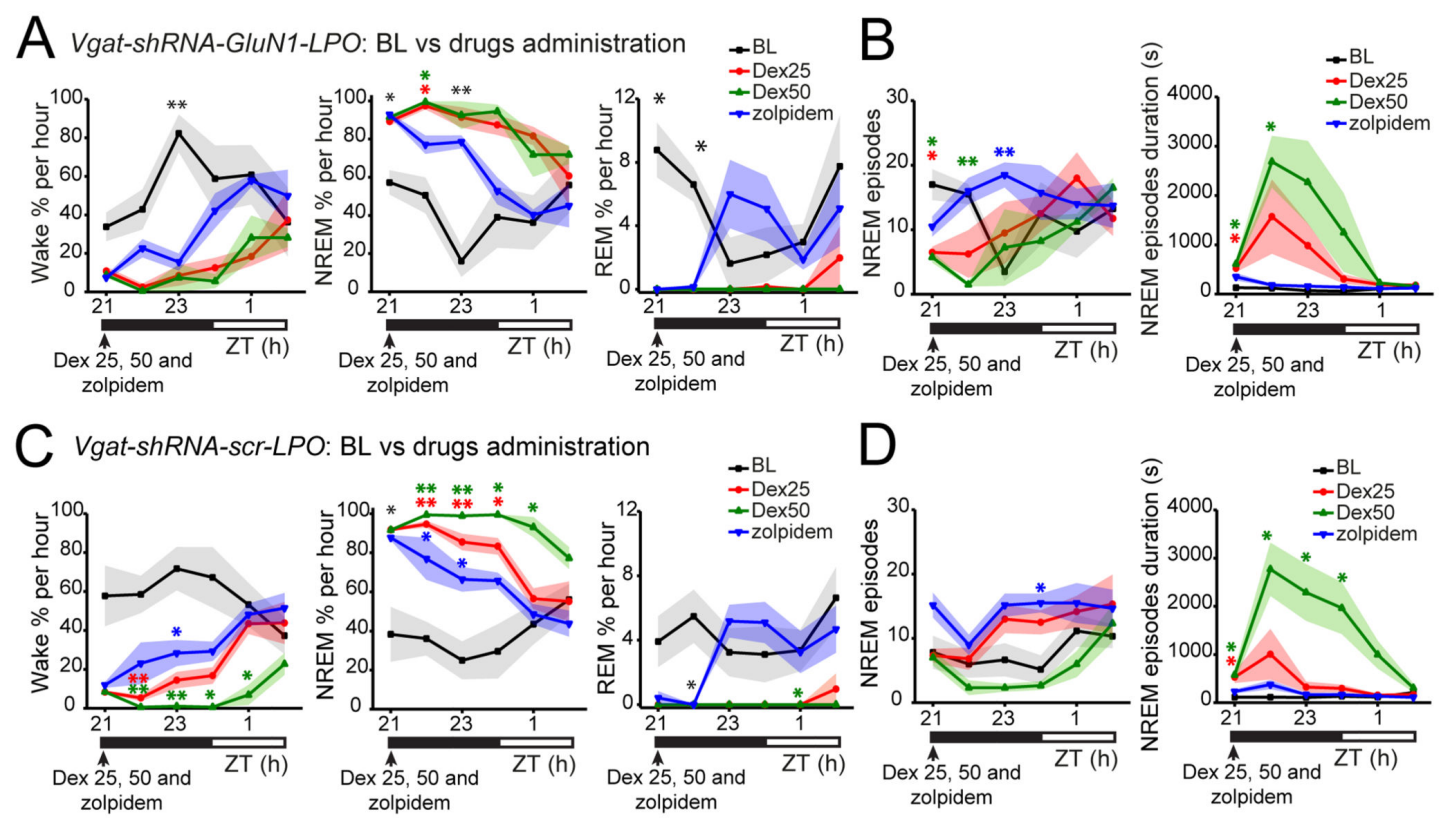
A sedative (Dex) and sleeping medication (zolpidem) reduce sleep fragmentation in *Vgat-shRNA-GluN1-LPO* mice. ***A***, *Vgat-shRNA-GluN1* mice BL vigilance state distribution as percentage of 1h in Wake (left), NREM (center) and REM sleep (right) compared with the distribution after injection at ZT21 of Dex 25 μg/kg (red), Dex 50 μg/kg (green) and zolpidem 5 mg/kg (blue). ***B***, *Vgat-shRNA-GluN1-LPO* number of NREM episodes (left) and NREM episode mean duration (right) comparing baseline to both Dex (25 μg/kg and 50 μg/kg) and zolpidem, ***C***, *Vgat-shRNA-Scr-LPO* mice BL states distribution as percentage of 1h in Wake (left), NREM (center) and REM sleep (right) compared with the distribution after injection at ZT21 of Dex 25 μg/kg (red), Dex 50 μg/kg (green) and zolpidem 5mg/kg (blue) at ZT21. ***D***, control *Vgat-shRNA-Scr-LPO* number of NREM sleep episodes (left) and NREM sleep episode mean duration (right) comparing BL to Dex (25 μg/kg and 50 μg/kg) and to zolpidem (5mg/kg). For panels **A** and **B**, *Vgat shRNA-GluN1-LPO, n* = 4. For panels **C** and **D**, *Vgat-shRNA-scr-LPO, n* = 6. Data in all panels are represented as mean ± SEM(*p < 0.05, ** *p* < 0.005).

Next, we tested the effects on sleep fragmentation in *Vgat-shRNA-GluN1-LPO* mice by using the sedative drug Dex and the sleep drug zolpidem. Both doses of Dex (25 and 50 μg/kg), and zolpidem (5 mg/kg), injected into *Vgat-shRNA-GluN1-LPO* mice reduced Wake and REM sleep times, increasing the time spent in NREM sleep (two-way repeated measures ANOVA and Dunnett’s *post-hoc* test: for Wake time x drug, *F_(15, 60)_* = 2.7, *p* = 0.003; time, *F_(2.7, 32)_* = 6.1, *p* = 0.0028; dose, *F_(3, 12)_* = 15.2, *p* = 0.0002; for NREM sleep time x drug, *F_(15, 60)_* = 3.1, *p* = 0.001; time, *F_(2.6, 31.5)_* = 7.8, *p* = 0.0008; dose, *F_(3,12)_* = 22.7, *p* < 0.0001; for REM sleep time x drug, *F_(15,60)_* = 2.9, *p* = 0.002; time, *F_(2.4,28.9)_* = 1.7, *p* = 0.2; dose, *F_(3, 12)_* = 20.2, *p* < 0.0001, *n* = 4 mice, Fig. 11A). Looking at the sleep architecture, Dex (25 and 50 μg/kg) transiently decreased the NREM sleep episode number, while zolpidem increased (two-way repeated measures ANOVA and Dunnett’s *post-hoc* test: time x drug, *F_(15, 60)_* = 2.5, *p* = 0.007; time, *F_(3.2, 38)_* = 1.7, *p* = 0.2; drug, *F_(3, 20)_* = 2.8, *p* = 0.08, Fig. 11B, left panel). Both doses of Dex increased the NREM average episode duration, while zolpidem had no effects in maintaining longer NREM sleep bouts (two-way repeated measures ANOVA and Dunnett’s *post-hoc* test: time x drug, *F_(15, 60)_* = 3.6, *p* = 0.0002; time, *F_(2, 24.3)_* = 8.3, *p* = 0.002; drug, *F_(3, 12)_* = 6.7, *p* =0.007, Fig. 11B, right panel). A similar effect was also present in control *Vgat-shRNA-scr-LPO* mice, where we observed an increase in NREM sleep time and a decrease in time mice spent awake and in REM sleep when mice were injected with Dex and zolpidem (two-way repeated measures ANOVA and Dunnett’s *post-hoc* test: for wake time x drug, *F_(15,100)_* = 2.7, *p* = 0.002; time, *F(3.6,72)* = 3.5, *p* = 0.01; dose, *F_(3,20)_* = 78.4, *p* < 0.0001; for NREM sleep time x drug, *F_(15, 100)_* = 3.2, *p* = 0.0003; time, *F_(3.7,74.2)_* = 4.8, *p* = 0.002; dose, *F_(3,20)_* = 107.6, *p* < 0.0001; for REM sleep time x drug, *F_(15,100)_* = 1.9, *p* = 0.03; time, *F_(3.2,65)_* = 1.9, *p* = 0.13; dose, *F_(3,20)_* = 34.6, *p* < 0.0001, n = 6 mice, Fig. 11C). As for *Vgat-shRNA-GluN1-LPO* mice, *Vgat-shRNA-scr-LPO* mice did not show changes in NREM sleep architecture after zolpidem injection. NREM sleep episodes only decreased with zolpidem (two-way repeated measures ANOVA and Dunnett’s *post-hoc* test: time x drug, *F_(15, 100)_* = 1.3, *p* = 0.2; time, *F_(3.4, 67.8)_* = 5.6, *p* = 0.001; drug, *F_(3, 20)_* = 11.8, *p* =0.0001, Fig. 11D, left panel), while NREM sleep mean duration briefly increased after Dex 25 μg/kg injection, and a more prolonged increase occurred when 50 μg/kg of Dex was injected (two-way repeated measures ANOVA and Dunnett’s *post-hoc* test: time x drug, *F_(15, 100)_* = 5.1, *p* < 0.0001; time, *F_(2.5, 497)_* = 8.7, *p* = 0.0002; drug, *F_(3, 20)_* = 20.6, *p* < 0.0001, Fig. 11D, right panel). Thus, sedatives and sleeping medications can transiently remove the insomnia (sleep-wake fragmentation) in mice lacking NMDA receptors in LPO GABA neurons.

## Discussion

The PO hypothalamus is required for both NREM and REM sleep generation and NREM sleep homeostasis (Nauta, 1946; McGinty and Sterman, 1968; Sherin et al., 1996; John and Kumar, 1998; Lu et al., 2000; Lu et al., 2002; Szymusiak et al., 2007; Zhang et al., 2015; Ma et al., 2019; Reichert et al., 2019). We explored how NMDA receptors on LPO neurons regulate sleep. Deleting the core GluN1 subunit of NMDA receptors from LPO neurons substantially reduced the excitatory drive onto these cells and abolished activity during all vigilance states. These *ΔGluN1-LPO* mice had less NREM sleep and altered REM sleep patterns (atonia was present, but there was reduced EEG theta activation). In addition, *ΔGluN1-LPO* mice had highly fragmented sleep-wake: they had many more episodes of wake and NREM sleep, but each episode was shorter. Thus, although *ΔGluN1-LPO* mice can still enter NREM sleep from wake, AMPA glutamate receptor excitation alone on LPO sleep-promoting neurons is insufficient to maintain NREM sleep or produce REM sleep. The *ΔGluN1-LPO* mice phenotype is quite similar (wake-NREM fragmentation, loss of REM sleep) to mice with a double (global) deletion of the muscarinic receptor genes *Chrm1* and *Chrm3* (Niwa et al., 2018), so this could intersect on the same pathway. The phenotype was further stratified. High sleep-wake fragmentation, but not sleep loss, was produced by selective GluN1 knock-down in GABAergic LPO neurons (*Vgat-shRNA-GluN1-LPO* mice).

The molecular mechanism of sleep homeostasis, whereby the time spent awake is tracked and leads to an increase drive to sleep, is not resolved. A mutation in one kinase, salt- inducible kinase3, which is expressed throughout the brain, reduces sleep homeostasis (Funato et al., 2016; Honda et al., 2018). In regions such as neocortex and hippocampus, increased time awake leads to increased phosphorylation of hundreds of synaptic proteins, including glutamate receptors (Wang et al., 2018; Bruning et al., 2019). Calcium entry through NMDA receptors has been suggested to be part of the sleep homeostasis mechanism that tracks time spent awake, and the calcium entry through NMDA receptors could stimulate phosphorylation (Liu et al., 2016; Tatsuki et al., 2016). But at least for the PO hypothalamus, which contains galanin neurons required for sleep homeostasis (Ma et al., 2019; Reichert et al., 2019), our findings show that NMDA receptors are not needed for sleep homeostasis.

The sleep homeostasis process is reflected in changes in EEG delta power (Borbely et al., 2016). During the 24-hour cycle, delta power is highest during the “lights on” sleep phase and declines as each NREM sleep bout progresses (Fig. 6D, E), which is thought to reflect the dissipation of the homeostatic sleep drive (Borbely et al., 2016). After sleep deprivation, neocortical activity in the subsequent (“recovery”) NREM sleep is deeper (more synchronized and thus has a higher delta power). However, we found that NMDA receptor deletion in LPO did not affect sleep homeostasis as defined by the classical criteria - EEG delta power showed its usual variation, an increase and decrease over 24 hours. Even placing *ΔGluN1-LPO* mice under high sleep pressure by sleep deprivation did not enable the mice to sleep well. The sleep fragmentation persisted even during the recovery sleep, and the fragmented sleep started with a higher delta power, as expected for recovery sleep in the sleep homeostasis model. So, the sleep homeostatic process seems independent of the mechanism maintaining consolidated sleep. In fact, during sleep deprivation, *ΔGluN1-LPO* mice made multiple attempted entries to sleep. It was as if the mice were chronically sleepy, but they nonetheless were not driven to sleep.

Many people suffer from occasional insomnia, but it can become a debilitating condition (Roth et al., 2011). Insomnia, as a clinical disorder, is defined as an inability to initiate or maintain sleep at least three times a week over three months, even when sleep conditions are otherwise optimal (Van Someren, 2020). Insomniacs frequently report that their sleep is non-restorative and that they sleep less. In fact, insomnia sufferers often have the same amounts of EEG-defined NREM sleep as controls, but oscillate frequently between wake and NREM sleep, so that their sleep is fragmented (Van Someren, 2020). The *Vgat-shRNA-GluN1-LPO* mice, which have the same amount of sleep, but high sleep-wake fragmentation, could be a useful model for intractable insomnia.

Behavioral therapy is often ineffective for treating intractable insomnia disorder, and medication remains an alternative approach if used cautiously (Shahid et al., 2012; An et al., 2020; Van Someren, 2020). Unlike sleep deprivation, which is usually efficient at inducing sleep, drugs could treat quite effectively the insomnia of *ΔGluN1-LPO* mice. Dexmedetomidine could transiently restore consolidated NREM sleep. Dexmedetomidine, an α2 adrenergic agonist, induces stage 3 NREM sleep in humans and NREM-like sleep in animals (Gelegen et al., 2014; Zhang et al., 2015; Akeju et al., 2018), and requires galanin/GABA neurons in LPO for its effects (Ma et al., 2019). Zolpidem (Ambien), a GABA_A_ receptor positive modulator, is a widely prescribed sleeping medication (Wisden et al., 2019). Its main effect in humans is to reduce latency to NREM sleep rather than maintaining consolidated sleep. Nevertheless, zolpidem did have a beneficial effect on both *ΔGluN1-LPO* and *Vgat-shRNA-GluN1-LPO* mice, restoring longer periods of NREM sleep.

Our findings also demonstrate a new aspect of REM sleep generation. REM sleep is characterized by a high theta:delta frequency ratio in the EEG together with muscle atonia. In rodents, the theta itself detected in the cortical EEG seems to originate mostly from the hippocampus. Indeed, the theta activation during REM is required for memory processing (Boyce et al., 2016; Izawa et al., 2019). Although the brainstem circuitry that generates muscle atonia during REM sleep is reasonably well understood, the circuitry that produces the theta activity in the EEG during REM sleep is only partially characterized, seeming to require distributed circuitry throughout the forebrain (Renouard et al., 2015; Peever and Fuller, 2016; Luppi et al., 2017; Izawa et al., 2019; Yamada and Ueda, 2019), including the MCH NREM-REM-promoting neurons in the lateral hypothalamus (Jego et al., 2013), REM-off and REM-on neurons in the dorsal medial hypothalamus (Chen et al., 2018), and GABA and cholinergic neurons in the medial septum that project to the hippocampus (Yoder and Pang, 2005). Although LPO is known to be required for REM sleep (Lu et al., 2000; Lu et al., 2002), we were surprised to discover that LPO neurons, regardless of type (*e.g. galanin, Vgat, Vglut2*), have actually their highest activity during REM sleep. We found that GluN1 knockdown in GABA and Vglut2 cells of LPO did not influence REM sleep, whereas the pan knockout in all LPO neurons did, so the cell type(s) expressing NMDA receptors responsible for REM sleep generation in LPO require further investigation.

We speculate that NMDA receptor properties could be responsible for maintaining NREM and REM sleep promoting LPO neurons in the “on” state. In contrast to AMPA-gated ionotropic glutamate receptors, NMDA receptors stay open for around 100 msec to 1 s, as well as having a voltage-dependent magnesium block (Paoletti et al., 2013). Because of these properties, NMDA receptors have been intensely studied for their role in synaptic plasticity. But these same properties also allow NMDA receptors to act as pacemakers, controlling rhythmic firing e.g. in those circuits involved in breathing, swimming and walking (Steenland et al., 2008; Li et al., 2010), as well as contributing to the generation of burst firing of reticular thalamic neurons (Deleuze and Huguenard, 2016). The long open times of NMDA receptors, especially those located extrasynaptically, could be contributing to tonic excitation (Sah et al., 1989; Neupane et al., 2021), stabilizing hypothalamic sleep-on neurons in their firing mode. It will be interesting to see if this role of NMDA receptors generalizes to other sleep-promoting circuits. For example, we previously found that genetic silencing of mouse lateral habenula neurons with tetanus toxin light-chain produced high NREM sleep-wake fragmentation with conserved amounts of total sleep and wake (Gelegen et al., 2018). It seems likely that disrupting NMDA receptors on these cells would also produce insomnia, given that that NMDA receptors are needed to keep lateral habenula cells in burst firing (active) mode (Yang et al., 2018; Cui et al., 2019).

In conclusion, we have found that selectively reducing NMDA receptors in the LPO hypothalamic area causes insomnia (wake-NREM sleep fragmentation) and loss of theta activity during REM sleep. Given that sleep homeostasis is intact in mice with no NMDA receptors in LPO hypothalamus, the mechanism of sleep maintenance is distinct from that of the sleep drive itself.

## Significance Statement

Insomnia is a common affliction. Most insomniacs feel that they do not get enough sleep, but in fact, often have good amounts of sleep. Their sleep, however, is fragmented, and sufferers wake up feeling unrefreshed. It is unknown how sleep is maintained once initiated. We find that in mice, NMDA-type glutamate receptors in the hypothalamus are the main drivers of excitation and are required for a range of sleep properties: they are, in fact, needed for both sustained NREM sleep periods, and REM sleep generation. When NMDA receptors are selectively reduced from inhibitory preoptic neurons, mice have normal total amounts of sleep but high sleep-wake fragmentation, providing a model for studying intractable insomnia.
